# The CD2v protein of African swine fever virus inhibits macrophage migration and inflammatory cytokines expression by downregulating EGR1 expression through dampening ERK1/2 activity

**DOI:** 10.1186/s13567-023-01239-w

**Published:** 2023-11-15

**Authors:** Min Zhang, Lilei Lv, Huaye Luo, Hongming Cai, Lingxue Yu, Yifeng Jiang, Fei Gao, Wu Tong, Liwei Li, Guoxin Li, Yanjun Zhou, Guangzhi Tong, Changlong Liu

**Affiliations:** 1 Shanghai Veterinary Research Institute, Chinese Academy of Agricultural Sciences, Shanghai, 200241 China; 2 College of Veterinary Medicine, Northeast Agricultural University, Harbin, 150030 China; 3 Key Laboratory of Fujian-Taiwan Animal Pathogen Biology, College of Animal Sciences, Fujian Agriculture and Forestry University, Fuzhou, 350002 China; 4 Jiangsu Co-Innovation Center for the Prevention and Control of Important Animal Infectious Disease and Zoonosis, Yangzhou University, Yangzhou, 225009 China

**Keywords:** ASFV, CD2v, cell migration, EGR1, ERK1/2, inflammatory cytokine

## Abstract

African swine fever virus (ASFV) is a highly contagious and deadly virus that leads to high mortality rates in domestic swine populations. Although the envelope protein CD2v of ASFV has been implicated in immunomodulation, the molecular mechanisms underlying CD2v-mediated immunoregulation remain unclear. In this study, we generated a stable CD2v-expressing porcine macrophage (PAM-CD2v) line and investigated the CD2v-dependent transcriptomic landscape using RNA-seq. GO terms enrichment analysis and gene set enrichment analysis revealed that CD2v predominantly affected the organization and assembly process of the extracellular matrix. Wound healing and Transwell assays showed that CD2v inhibited swine macrophage migration. Further investigation revealed a significant decrease in the expression of transcription factor early growth response 1 (EGR1) through inhibiting the activity of extracellular signal-regulated kinase 1 and 2 (ERK1/2). Notably, EGR1 knockout in swine macrophages restricted cell migration, whereas EGR1 overexpression in PAM-CD2v restored the ability of macrophage migration, suggesting that CD2v inhibits swine macrophage motility by downregulating EGR1 expression. Furthermore, we performed chromatin immunoprecipitation and sequencing for EGR1 and the histone mark H3K27 acetylation (H3K27ac), and we found that EGR1 co-localized with the activated histone modification H3K27ac neighboring the transcriptional start sites. Further analysis indicated that EGR1 and H3K27ac co-occupy the promoter regions of cell locomotion-related genes. Finally, by treating various derivatives of swine macrophages with lipopolysaccharides, we showed that depletion of EGR1 decreased the expression of inflammatory cytokines including TNFα, IL1α, IL1β, IL6, and IL8, which play essential roles in inflammation and host immune response. Collectively, our results provide new insights into the immunomodulatory mechanism of ASFV CD2v.

**Supplementary Information:**

The online version contains supplementary material available at 10.1186/s13567-023-01239-w.

## Introduction

African swine fever (ASF) is a fatal disease that affects not only domestic pigs but also wild boars. It is caused by the African swine fever virus (ASFV), the sole member of the *Asfarviridae* family. ASF is a major threat to domestic pigs, as it lowers production, leading to severe economic losses in pig-producing countries. As of now, there is no cure or licensed vaccine for this disease [1, 2]. The ASFV genome is a large double-stranded DNA molecule (170–193 kbp) encoding 151–167 proteins, which are crucial for viral replication and assembly, as well as in modifying host cellular functions and evading the immune system [1].

ASFV exhibits a highly limited cellular tropism, with replication primarily occurring in mononuclear phagocytic cells such as monocytes, macrophages, and dendritic cells. These cells are essential for the initiation of innate immune responses, as well as presenting antigens to T cells, thereby triggering an adaptive immune response [3–5]. During infection, ASFV expresses multiple proteins that disrupt various host pathways ultimately promoting efficient viral replication [4].

The ASFV *EP402R* gene encodes CD2v, a type I transmembrane protein that resembles the host adhesion molecule CD2 expressed on T and NK cells. CD2v is located on the external membrane layer of ASFV particles and is required for the virus-induced rosetting of erythrocytes around infected cells. CD2v comprises a signal peptide, transmembrane domain, and 147-amino acid cytoplasmic tail [6, 7]. The cytoplasmic tail of CD2v has the ability to bind to the cytoplasmic adaptor protein SH3P7, which is involved in multiple cellular functions, including signal transduction and vesicle transport [8]. Moreover, during ASFV infection, CD2v can interact with the adaptor protein AP-1. However, the function of the CD2v-AP-1 complex remains unknown [9].

CD2v has been found to play an important role in virulence, in the induction of protective immune responses, and in host immunomodulation. Deletion of CD2v in the European isolate BA71 led to virus attenuation and induced protection against virulent virus challenge [10]. In contrast, CD2v deletion in virulent strains such as Malawi and Georgia 2007 did not result in viral attenuation in swine [11, 12]. Recombinant pseudorabies virus-mediated expression of CD2v can stimulate the production of anti-CD2v specific antibodies and trigger a specific cellular immune response in mice [13]. According to a recent study, there are six T-cell epitopes found in CD2v and C-type lectin (EP153R) [14]. Moreover, during ASFV infections, the CD2v and/or C-type lectins play a vital role in providing protection against homologous ASFV infection [15]. Deleting *CD2v* and *EP153R* genes from the virulent strain Georgia abolishes its effectiveness as a vaccine [16]. However, the double deletion of *CD2v* and *UK* genes from ASFV strain SY18 did not affect viral replication in primary porcine alveolar macrophages, but it induced ASFV-specific antibodies that guarded pigs against the challenge with parental SY18 strain [17]. Although CD2v is not crucial for viral replication in pigs, it plays a vital role in viral replication in the tick vector by enhancing viral uptake across the tick midgut cycle [18].

The infection of cultured peripheral blood mononuclear cells with ASFV leads to a reduction in the mitogen-dependent proliferation of uninfected lymphocytes. However, the elimination of the gene that encodes CD2v abolishes this effect, suggesting that CD2v plays a part in immunosuppression [12]. During an acute ASFV infection, excessive standby lymphocytes depleted in lymphoid tissues are observed in pigs [19, 20]. Factors that are released or secreted by infected macrophages are likely to trigger this process [21–23]. A study conducted recently has shown that CD2v, which is present on the infected cell membranes or released from infected macrophages, interacts with nearby lymphocytes and macrophages through CD58. This interaction leads to the activation of NF-kB, which, in turn, induces apoptosis of both lymphocytes and macrophages [24]. The mechanism responsible for the release of CD2v is yet to be determined. It is possible that CD2v is secreted or leaves the cell through membrane blebbing or exosomes. Further research is needed to fully understand the release mechanism of CD2v.

The transcription factor EGR1, also known as Krox24 and ZNF225, belongs to the immediate early response genes family. This protein contains a conserved zinc finger DNA-binding domain and binds to the GCG(G/T)GGGCG region of target gene promoters [25]. Various stimuli, such as growth factors, cytokines, mitogens, and stress, activate EGR1 expression through different mitogen-activated protein kinase pathways (MAPKs) including the extracellular signal-regulated kinase (ERK), p38 MAPK pathways, and c-Jun NH2-terminal kinase (JNK) [26, 27]. EGR1 plays a significant role in a multitude of biological processes. It participates in various host signal transduction cascades that regulate crucial cellular activities such as mediating cell proliferation, differentiation, survival, and apoptosis.

As a transcription factor, EGR1 is responsible for inducing the expression of several cytokine genes like TNFα, IL2, and ICAM-1 [28–30]. These cytokines are important regulators of immune response. Additionally, EGR1 serves a critical function in inflammation by regulating the expression of chemokines, adhesion receptors, and pro-coagulant genes [31]. Furthermore, EGR1 is involved in thymocyte development [32, 33]. It is also a necessary factor for macrophage-lineage differentiation [34].

Moreover, EGR1 has been implicated in viral infection. The foot-and-mouth disease virus (FMDV) infection induces the expression of EGR1 in porcine cells, resulting the suppression of FMDV replication [35]. In murine coronavirus infection, EGR1 expression is dramatically increased and suppresses the expression of BNip3 to prevent apoptosis of astrocytoma cells [36]. During infection with Venezuelan equine encephalitis virus (VEEV), EGR1 plays a role in regulating pro-apoptotic pathways, ultimately contributing to VEEV-induced cell death [37]. In addition, EGR1 promotes the replication of Enterovirus 71 by directly binding to viral genome RNA [38]. In contrast, in the case of neural dysfunction induced by simian immunodeficiency virus, the expression of EGR1 is downregulated rather than upregulated [39].

In this study, we aimed to identify the genes and biological processes regulated by ASFV CD2v in swine macrophages to determine their role in regulating the immune response. To determine the CD2v-dependent transcriptome, we conducted RNA-seq analysis and performed gene ontology (GO) terms enrichment analysis and gene set enrichment analysis (GSEA). Our results demonstrated that CD2v modulated the extracellular matrix organization and assembly. Wound healing and Transwell assays showed that CD2v inhibited swine macrophage migration. Notably, we found that CD2v inhibited swine macrophage migration by downregulating the expression of the transcription factor EGR1 via inhibiting the activity of ERK1/2. Next, the cistrome of EGR1 in swine macrophages was defined using ChIP-seq. Our findings showed co-occupancy of EGR1 and H3K27 acetylation (H3K27ac) on the promoter regions of cell locomotion-related genes. Furthermore, we found that CD2v inhibited the expression of inflammatory cytokines by downregulating the expression of EGR1 in swine macrophages. Overall, our investigation has shed light on the molecular events by which CD2v regulates swine macrophage migration and inflammatory cytokines through EGR1 downregulation by dampening ERK1/2 activity.

## Materials and methods

### Cell culture

The immortalized porcine alveolar macrophages 3D4/21 (CRL-2843) were purchased from the ATCC (American Type Culture Collection) and maintained in RPMI 1640 medium (Gibco), supplement with 10% fetal bovine serum (FBS) (Gibco), 100U/mL penicillin & 100 µg/mL streptomycin. Lenti-X™ 293 T cells (Clontech) and HeLa cells were cultured in DMEM (Gibco) with 10% FBS, 100U/mL penicillin & 100 µg/mL streptomycin.

### Lentiviral vector construction

The coding sequence (EP402R gene) of CD2v from the ASFV-SY18 strain (GenBank: MH766894.) with a C-terminal Flag tag flanked by BamHI and EcoRI restriction sites was optimized for codon usage, chemically synthesized, and inserted into the lentiviral vector pLV-EF1a-IRES-Hygro (Addgene, Cat#:85134) using a Hi-T4 DNA Ligase Kit (NEB, Cat#: M2622S). Similarly, the sequence encoding EGFP (enhanced green fluorescent protein) was amplified with primers and inserted into the same vector pLV-EF1a-IRES-Hygro. For the EGR1 expression vector, the sequence encoding porcine EGR1 (GenBank: XM_003123974.6) flanked by BamHI and EcoRI restriction sites was synthesized and inserted into the lentiviral vector pLV-EF1a-IRES-Puro (Addgene, Cat#:85132). EGFP was amplified using primers and cloned into pLV-EF1a-IRES-Puro using the same procedure. The vectors were transformed into Stbl3 cells (Thermo Fisher), and the bacteria were growing at 37 °C on LB Agar plates that contained ampicillin (100 mg/mL). Colonies were selected and cultured overnight at 37 °C in LB medium with ampicillin (100 mg/mL). Plasmids were extracted and the sequences were confirmed by DNA sequencing.

### Lentiviral vector production and titration

Lentivirus packaging and titration were performed as previously described [40, 41]. Briefly, Lenti-X™ 293 T cells were seeded at a density of 1.2 × 10^7^ per T175 flask. The next day, a total of 60 μg of plasmid DNA was introduced into the cells. The DNA mixture included 30 μg of transgene vector plasmid, 19.5 μg of pSPAX2 (Addgene, Cat#:12260), and 10.5 μg of pMD2.G (Addgene, Cat#:12259), using the CaCl2 method. After 48 h of transfection, the virus-containing supernatants were collected and filtered through a 0.45 μm filter (Millipore, Cat#: SCHVU01RE). Subsequently, the virus supernatants were concentrated and divided into aliquots for storage at −80 °C.

The lentiviral titers were determined using previously described protocol [42]. Briefly, HeLa cells were placed in a 6-well plate at a density of 1 × 10^6^ cells per well. After 8 h of plating, various amounts of viruses were introduced into the wells along with 8 μg/mL polybrene. At 24 h after transduction, cells in each well of the 6-well plate were split into duplicate wells. One replicate received either 500 μg/mL Hygromycin B or 2.5 μg/mL Puromycin. After 3 days (or as soon as no surviving cells remained in the no-transduction control under drug selection), cells were counted to calculate percent transduction. Percent transduction is calculated as cell count from the replicate with puromycin divided by cell count from the replicate without puromycin multiplied by 100.

### Virus transduction and cell line establishment

PAMs were seeded in a six-well plate with 2 × 10^5^ cells per well. Twelve hours after seeding, 5 MOI of CD2v or EGFP lentiviruses were used to infect the cells with 8 μg/mL polybrene. The medium was refreshed 24 h after transduction. 48 h after transduction, the cell culture medium was substituted with the medium that contained 500 μg/mL hygromycin B for one week. Positive cells were collected and subcultured.

### EGR1 knockout in PAMs

EGR1 knockout PAMs were generated using CRISPR/Cas9 as previously described [40, 41]. Briefly, a pair of small guide RNAs (Additional file 11) was designed using the online tool E-CRISP, targeting exon 1 of the porcine *EGR1* gene (Entrez gene ID: 100520726) and cloned into LentiCRISPRv2-puro (cat#:98290, Addgene) with the BsmbI cloning site. PAMs were transfected with the two *EGR1* small guide RNA (sgRNA) plasmids using Lipofectamine 3000 (Life Technologies). At 24 h after transfection, the supernatant was substituted with the medium that contained 2.5 μg/mL puromycin and was then incubated for 3 days. Subsequently, single cells were diluted manually into 96-well plates. The single-cell clones were then expanded and confirmed by Western blotting and PCR using the primers shown in Additional file 11. The DNA sequence across the deleted region was amplified from the genomes of EGR1-KO and EGR1-WT PAM lines. The PCR products were then sequenced to confirm the deletion of the *EGR1* gene.

### EGR1 overexpression in PAM-CD2v cells

PAM-CD2v cells were seeded at a density of 2 × 10^5^ cells per well in a six-well plate. Twelve hours after seeding, 5 MOI of EGR1 or EGFP lentiviruses were used to infect the cells with 8 μg/mL polybrene. The medium was refreshed at 24 h post transduction. 48 h after transduction, the cell culture medium was replaced with fresh medium with puromycin at concentration of 2.5 μg/mL for 1 week. The positive cells were collected and cultured.

### Western blotting

The cells were rinsed twice with ice-cold DPBS and then lysed in RIPA buffer containing a cocktail of protease inhibitors (cat#: P8340, Sigma). The protein concentrations in the lysates were determined using a Pierce BCA Protein Assay Kit (cat#: 23227, Thermo Scientific). Approximately 20 μg of denatured lysates were subjected to electrophoresis on a 10% SDS-PAGE gel and subsequently transferred to nitrocellulose membranes. The membranes were blocked with 5% non-fat dry milk in TBST (10 mM Tris–HCl, pH 7.5, 150 mM NaCl, and 0.1% Tween-20) for 1 h at room temperature (RT). Then, the membranes were incubated with the primary antibody overnight at 4 °C. After washing with TBST, the blots were incubated with a horseradish peroxidase (HRP)-conjugated secondary antibody for 1 h at RT and washed with TBST twice. Finally, the signals were detected using a ChemiDoc™ MP imaging system (Bio-Rad) after incubation with SuperSignal™ West Pico Chemiluminescent substrate (cat#: 34077, Thermo Scientific). The primary antibodies used included GAPDH (1:1000, cat#: 5174, CST), flag-tag (1:1000, cat#: 8146, CST), EGR1 (1:1000, cat#: 4154, CST), ERK1/2 (1:1000, cat#: 9102, CST), phoshoERK1/2 (1:1000, cat#: 9101, CST), beta Tubulin (1:10 000, Cat#: 66240, Proteintech). Horseradish peroxidase-linked secondary antibodies for rabbit IgG (1:2000, cat#: 7074, CST) and mouse IgG (1:2000, cat#: 7076, CST) were used.

### Cell proliferation assays

PAM-GFP, PAM-CD2v, EGR1-WT PAMs, and EGR1-KO PAMs were seeded at 2,000 cells per well in a 96-well plate. Eight replicates were performed for each cell line. On day 1 and 2, 700 μM resasurin (Sigma) in DPBS was added into each well to a final concentration of 35 μM. The cells were incubated for 4 h at 37 °C_._ Fluorescence was then measured using a Biotek Epoch with 560 nm emission/590 nm excitation filters.

### Erythrocyte adsorption of CD2v

PAM-CD2v and PAM-GFP macrophages were suspended in RPMI 1640 at a concentration of 10^7^ cells/mL. Approximately 5 × 10^6^ cells (500 μL) and an equal volume of 1% porcine erythrocyte suspension were then transferred to a 1.5 mL tube and incubated at 37 °C for 5 min. The cells were pelleted at 500 rpm for 5 min and then incubated at 4 °C for 2 h or overnight. The supernatant was discarded, 4% paraformaldehyde was added to the cells and then fixed at RT for 10 min. The cells were observed and photographed under a Model Eclipse Ts2R-FL microscope (Nikon).

### RT-qPCR

The RNA was extracted from the cells using an RNA Easy Fast Tissue/Cell Kit (cat#: DP451, Tiangen). Subsequently, cDNA was synthesized using a PrimeScript™ RT reagent Kit (cat#: RR047A, Takara) with 1 μg of total RNA. For the qPCR analysis, the KAPA SYBR Fast Universal qPCR Master Mix (cat#: KK4602, Kapa Biosystems) was used along with a Roche LightCycler96 PCR System instrument. The relative gene expression levels were determined using GAPDH as a reference. The primer sequences can be found in Additional file 11.

### Immunostaining and microscopy

The cells were plated in a 6-well plate at a density of 1 × 10^5^ cells per well and allowed to grow until reaching 50% confluence. Subsequently, the cells were fixed with 4% paraformaldehyde for 45 min at room temperature (RT). Permeabilization was carried out using 0.25% Triton X-100 for 20 min. After washing twice with DPBS, the cells were blocked with 5% BSA for 30 min at RT. Next, the cells were incubated with the primary antibody at 37 °C for 1 h, followed by incubation with a secondary antibody, goat anti-mouse IgG (H + L) cross-adsorbed, DyLight 594 (1:1000, Cat# 35511, Invitrogen) for 45 min at 37 °C. The cell nuclei were counterstained with DAPI (cat#: D9542, Sigma) at RT for 5 min. Fluorescent images were captured using a fluorescence microscope BZ-X800E (Keyence).

### LPS treatment

Various swine PAM derivatives were plated in 6-well plates. After reaching 80% confluence, the cells were treated with 2 μg/mL LPS for 4 h. After treatment, the cells were harvested, and total RNAs were purified for further analysis.

### Inhibition of ERK1/2 activity in PAMs

The PAMs were plated in 6-well plates. After reaching 80% confluence, the ERK1/2 inhibitor, FR180204 (cat#: HY-12275, Sigma), was added to the cells. The same volume of DMSO was used as the control. The PAMs were treated for 48 h, and the proteins were isolated and analyzed by Western blotting.

### Transwell migration assay

Transwell chambers were placed into a 24-well plate, and 600 μl of DMEM containing 20% FBS was added to the lower compartment. In the upper compartment, the cells were trypsinized, suspended, and seeded at a density of 8 × 10^3^ cells per chamber in 200 μL of serum-free RPMI 1640. The cells were then incubated for either 24 h or 48 h. After incubation, the Transwell chambers were removed, washed twice with DPBS, and transferred to a new 24-well plate. The upper side of the membrane was gently swabbed using a cotton swab to remove any remaining cells. The cells that had migrated and reached the lower chamber were fixed with methanol and stained with 0.5% (w/v) crystal violet. Images of the migrated cells were captured using an Eclipse Ts2R-FL microscope (Nikon, Japan). The number of attached and migrated cells was determined by capturing five random fields and analyzing the images using ImageJ software. The cell numbers are presented as the average staining area per image.

### Wound-healing assay

Cells were trypsinized and resuspended in the medium, then transferred to 6-well plates and allowed to grow to confluence. The supernatant was replaced with a medium without FBS. Subsequently, linear wounds were scraped into the cell monolayer with pipette tips. The debris was eliminated, and the wound edge was smoothed by washing the cells once. The cells were then incubated in serum-free medium, and the wound was observed and photographed at 0 h, 12 h, and 24 h after incubation using a Model Eclipse Ts2R-FL microscope (Nikon, Japan). The width of the wound was measured with the ImageJ software. The relative wound size of cells was normalized to 0 h.

### ChIP-seq and analysis

PAMs were seeded in a 150 mm dish at a density of 1 × 10^7^ cells per dish with 20 mL of medium. Chromatin immunoprecipitation (ChIP) was performed using the SimpleChIP Plus Enzymatic Chromatin IP Kit (cat#:9005, CST) following the manufacturer’s protocol. Briefly, approximately 60 million cells were cross-linked by treating them with 540 µL of 37% formaldehyde in 20 mL medium, followed by incubation at room temperature (RT) for 10 min. The formaldehyde cross-linking was then quenched using 1 mL of 2.5 M glycine at RT for 5 min. The cells were subsequently washed twice with cold DPBS and transferred to a 15 mL conical tube. The cells were resuspended in 1 mL of cold buffer A, supplemented with a proteinase inhibitor cocktail and dithiothreitol (DTT), and incubated on ice for 10 min. Afterward, the cells were centrifuged at 2000 *g* for 5 min at 4 °C to pellet the nuclei. The resulting pellet was resuspended in the ChIP lysis buffer (150 mM NaCl, 0.7% SDS, 500 mM DTT, 5 mM EDTA, and 1% Triton X-100). To achieve an average chromatin fragment length of 200–600 bp, the chromatin was digested with micrococcal nuclease (cat#: 10,011, CST) at 37 °C for 20 min using a Scientz-950E ultrasonicator (Ningbo Scientz Biotech, Ningbo, China). The chromatin lysate was then diluted fivefold with SDS-free ChIP lysis buffer (cat#: 7008, CST), and 10 μg of the EGR1 antibody (1:50, cat#: 4154, CST), H3K27ac antibody (1:100, cat#: 8173, CST), or IgG antibody (1:100, cat#: 2729, CST) was added to the 20 million cell lysates along with ChIP-Grade Protein G Magnetic Beads (cat#: 9006, CST). The mixture was incubated overnight at 4 °C. On the second day, the beads were washed twice using low-salt and high-salt solutions and then resuspended in ChIP Elution Buffer (cat#: 7009, CST). The beads were incubated at 65 °C for 30 min to elute the complexes and reverse the cross-linking. Following treatment with proteinase K (0.5 mg/mL) for 2 h, the DNA was purified using centrifugal columns. The ChIP-seq libraries were prepared according to the Illumina ChIP-seq library preparation protocol, and the libraries were sequenced on an Illumina NovaSeq 6000 platform. The raw reads were processed and aligned to the Susscrofa 11 reference genome using BWA. Peak calling was performed using HOMER, and the annotation was conducted using the ChIPseeker package [43]. The processed data were visualized using the Integrative Genomics Viewer and the EnrichedHeatmap package [44].

### RNA-seq and analysis

RNA-sequencing and subsequent data analysis were performed following previously described methods [45]. Briefly, total RNA was isolated from PAM-CD2v and PAM-GFP using the miRNeasy Mini Kit (Qiagen, Shanghai, China), and the quality of RNA was assessed using an Agilent Bioanalyzer (Agilent, Beijing, China).Libraries were prepared using the TruSeq Stranded mRNA Library Preparation Kit (Illumina, Shanghai, China) with 1 μg of total RNA, according to the manufacturer’s instructions. Sequencing was carried out on an Illumina NovaSeq 6000 instrument (Illumina, San Diego, CA, USA). The raw RNA-seq data were processed and aligned to the Susscrofa reference genome (Sus scrofa11) using TopHat software. The reads that were mapped uniquely were assembled into genes using the Ensembl Susscrofa annotation file. DESeq2 [46] was used to identify differentially expressed genes between PAM-CD2v and PAM-GFP, considering a significance level of *P* < 0.05, unless otherwise stated. Pearson’s coefficient was calculated using the cor function with the default parameters in R Hierarchical clustering analysis of gene expression patterns was performed using the ComplexHeatmap package [47]. Heatmaps based on the z-scores of the FPKM values were also generated using the ComplexHeatmap package. Gene set enrichment analysis [48] was conducted using GSEA 4.1.0 software with ranked log2FoldChange of annotated genes from the DESeq2 output and the standard weighted enrichment statistic against gene sets derived from the GO Biological Process ontology was used. Online software gprofiler was used to do GO term enrichment analysis.

### Statistical analysis

The data analysis was conducted using either R or GraphPad Prism 9 (GraphPad, San Diego, CA, USA). Unless stated otherwise, the data are expressed as the mean ± standard deviation (SD) of at least three replicates. The unpaired Student’s *t*-test was performed to calculate the *P*-values. The significance level (*P*-value) was set at < 0.05 (*), < 0.01 (**), and < 0.001 (***).

## Results

### Generation and characterization of ASFV CD2v-expressing stable macrophage cell line

CD2v is a structural transmembrane glycoprotein that is expressed on the surface of macrophages that have been infected with ASFV [6]. To explore the function of CD2v in porcine macrophages, stable CD2v-expressing PAMs were generated. We cloned codon-optimized EP402R into a lentiviral vector that encoded a Hygromycin B phosphotransferase gene linked to the EP402R gene by the IRES element. We also constructed a vector expressing enhanced GFP as a control (Additional file 1). We produced replication-defective lentiviral particles and infected PAM cells with 5.0 MOI to generate pools of CD2v- and GFP-overexpressing cells (designated PAM-CD2v and PAM-GFP, respectively). Two days after infection, positive cells were chosen through cultivation in the presence of 500 μg/mL Hygromycin B for 7 days.

**Figure 1:**
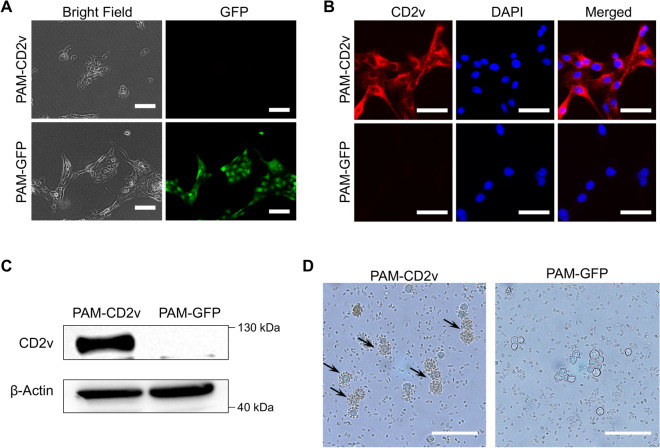
**Generation and Characterization of ASFV CD2v-expressing stable macrophage cell line.** **A** Morphology of PAM-CD2v and PAM-GFP cell lines. Scale bars: 200 μm. **B** Representative images for immunofluorescence staining of Flag tag for CD2v in PAM-CD2v and PAM-GFP cell lines. Scale bars: 200 μm. **C** CD2v protein levels were determined by Western blotting. **D** PAM-CD2v macrophages hemadsored swine red blood cells (RBCs). PAM-CD2v and PAM-GFP macrophages were subjected to overnight incubation with swine RBCs, followed by examination through light microscopy. The arrowheads indicate CD2v-dependent rosetting. Scale bars: 1000 μm.

The viability and morphology of PAM-CD2v cells were comparable to that of PAM-GFP cells (Figure 1A). Immunofluorescence staining showed that PAM-CD2v cells expressed high levels of the CD2v protein (Figure 1B). The majority of CD2v protein was detected in the cell membrane and perinuclear region (Figure 1B), which is in agreement with prior investigations of CD2v expression in macrophages infected with ASFV [6, 8, 24, 49]. The expression of CD2v protein in PAM-CD2v cells was confirmed through Western blotting (Figure 1C). The CD2v is responsible for erythrocyte hemadsorption in cells infected with ASFV [6, 7]. Hemadsorption tests were performed on both PAM-CD2v and PAM-GFP, and rosette formation was visualized to determine whether the PAM-CD2v cells were hemadsorbed. The results showed that PAM-CD2v had hemagglutination activity (Figure 1D), suggesting that the recombinant CD2v protein in swine macrophages was functional.

### CD2v affects key biological processes in swine macrophage

After establishing the CD2v-expressing swine macrophage line PAM-CD2v, we investigated the impact of CD2v on the biological function of swine macrophages. To identify the functional molecules and signaling pathways related to ASFV CD2v, we conducted transcriptome profiling based on RNA sequencing of PAM-CD2v and PAM-GFP lines with triplicate samples. Each sample obtained over 36 million reads that were uniquely mapped (Additional file 2A). The samples correlation heatmap demonstrated that PAM-CD2v and PAM-GFP lines were closely clustered (Additional file 2B). In line with the sample correlation analysis, the principal component analysis indicated that the transcriptomes of PAM-CD2v and PAM-GFP were distinguishable (Additional file 2C).

**Figure 2:**
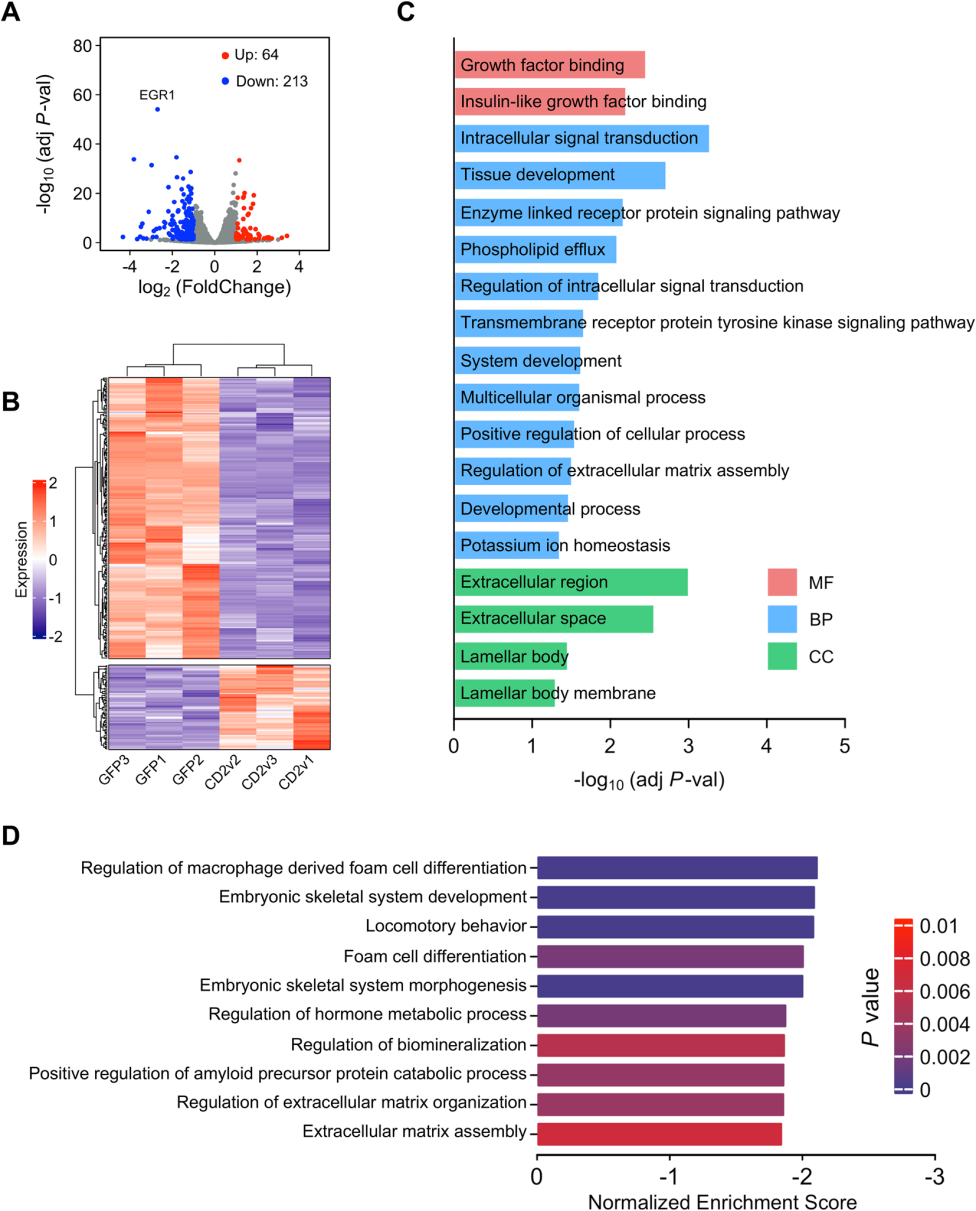
**Analysis of RNA-seq data**. **A** Volcano plot showing the RNA-seq expression analysis of PAM-CD2v compared to PAM-GFP. The plots illustrate the relationship between -log_10_ adjusted *P*-value and log_2_foldchange. Genes exhibiting ≥ twofold absolute value fold change meeting a 0.01 adjusted *P*-value threshold are highlighted in red and blue. **B** Heatmap and dendrogram illustrating the expression of all 64 upregulated and 213 downregulated genes. Expression: z-score of FPKM in each sample. **C** Histogram plot for molecular function (MF), biological process (BP), and cellular compartment (CC) gene ontology terms enriched in DEGs upon CD2v overexpressing in swine macrophage. **D** Histogram plots show the GSEA of the top 10 biological processes that are negatively regulated by CD2v protein.

We initially assessed the overall changes in gene expression between the two cell lines (Additional file 6) and identified 277 genes that exhibited significant differential expression (adjusted *P* < 0.05 and |log_2_FoldChange|> 1). Out of these genes, we found 64 to be upregulated and 213 to be downregulated in PAM-CD2v cells compared to PAM-GFP cells (Figure 2A). By performing unsupervised hierarchical cluster analysis on these differentially expressed genes (DEGs), we observed high agreement between triplicate cell lines and two broad categories of genes, those that were activated and those that were repressed (Figure 2B).

We then conducted a gene ontology (GO) enrichment analysis for all DEGs and found that the enriched terms (adjusted *P* < 0.05) were mainly related to growth factor binding, intracellular signal transduction, extracellular matrix assembly, extracellular region, and extracellular space (Figure 2C). Following this, we performed gene set enrichment analysis (GSEA) for all genes expressed in PAM-CD2v and PAM-GFP based on the biological process (BP) dataset. The significantly enriched pathways (*P* < 0.01, FDR < 25%) that were negatively regulated by CD2v included regulation of macrophage-derived foam cell differentiation, locomotor behavior, extracellular matrix organization, and extracellular matrix assembly (Figure 2D and Additional file 7). Taken together, these results suggest that ASFV CD2v regulates biological processes in swine macrophages.

### The CD2v protein of the African Swine Fever virus restricts the migration of swine macrophage

**Figure 3:**
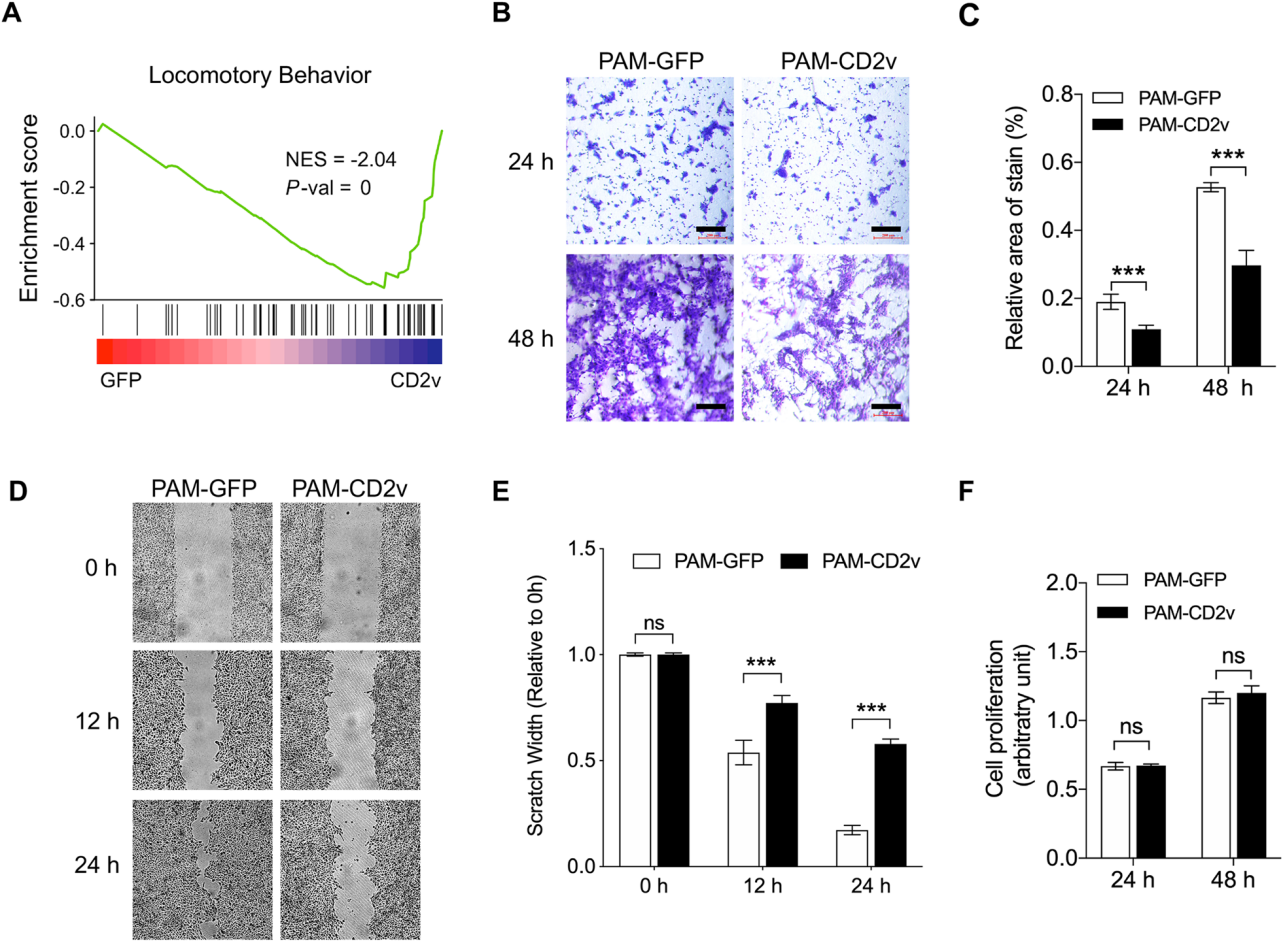
**CD2v protein inhibits swine macrophage migration**. **A** Gene set enrichment analysis (GSEA) plot of locomotory behavior gene set. Rank ordered by log_2_Foldchange of PAM-CD2v versus PAM-GFP. **B** Representative images of PAM-GFP and PAM-CD2v migration at 24 h and 48 h. Scale bar: 200 μm. **C** The number of migratory cells was evaluated by five random fields’ relative staining areas (***, *P* < 0.001, *n* = 5). **D** After an initial scratch, representative images of PAM-GFP and PAM-CD2v cells at 12 and 24 h. **E** Quantification of scratch width in PAM-GFP and PAM-CD2v macrophages (***, *P* < 0.001; ns, not significant; *n* = 6 per group). (F) Cell proliferation of PAM-GFP and PAM-CD2v macrophages was determined by resazurin at different time points as indicated (ns, not significant; *n* = 8).

GO term enrichment analysis showed that CD2v affected extracellular matrix assembly and organization. In addition, the GSEA results showed that CD2v negatively regulated the locomotor behavior of PAMs (Figure 3A). Almost all cells are capable of migration, but this is particularly important for cells participating in immune functions. To evaluate the contribution of CD2v in regulating swine macrophage migration in vitro, we performed transwell migration and wound-healing assays on both PAM-CD2v and PAM-GFP cells to investigate whether CD2v affects the migration of macrophages. The results of the transwell migration assay showed decreased migration of PAM-CD2v cells compared to control PAM-GFP cells, suggesting that CD2v has an inhibitory effect on swine macrophages (Figures 3B and C). CD2v restricting macrophage migration was also confirmed by a wound-healing assay (Figure 3D). The relative size of the wound in PAM-CD2v cells, normalized to the wound size at 0 h, was significantly higher than that of control PAM-GFP cells at 12- and 24-h time points (Figure 3E). Since the transwell migration and wound-healing assays might be biased by cell proliferation, cell proliferation was evaluated by resazurin assay. The results showed that CD2v expression had no impact on macrophage proliferation over 2 days monitoring period (Figure 3F). Altogether, these data indicate that CD2v expression reduces the migration of swine macrophages in vitro.

### CD2v downregulates EGR1 by inhibiting the activity of ERK1/2

**Figure 4:**
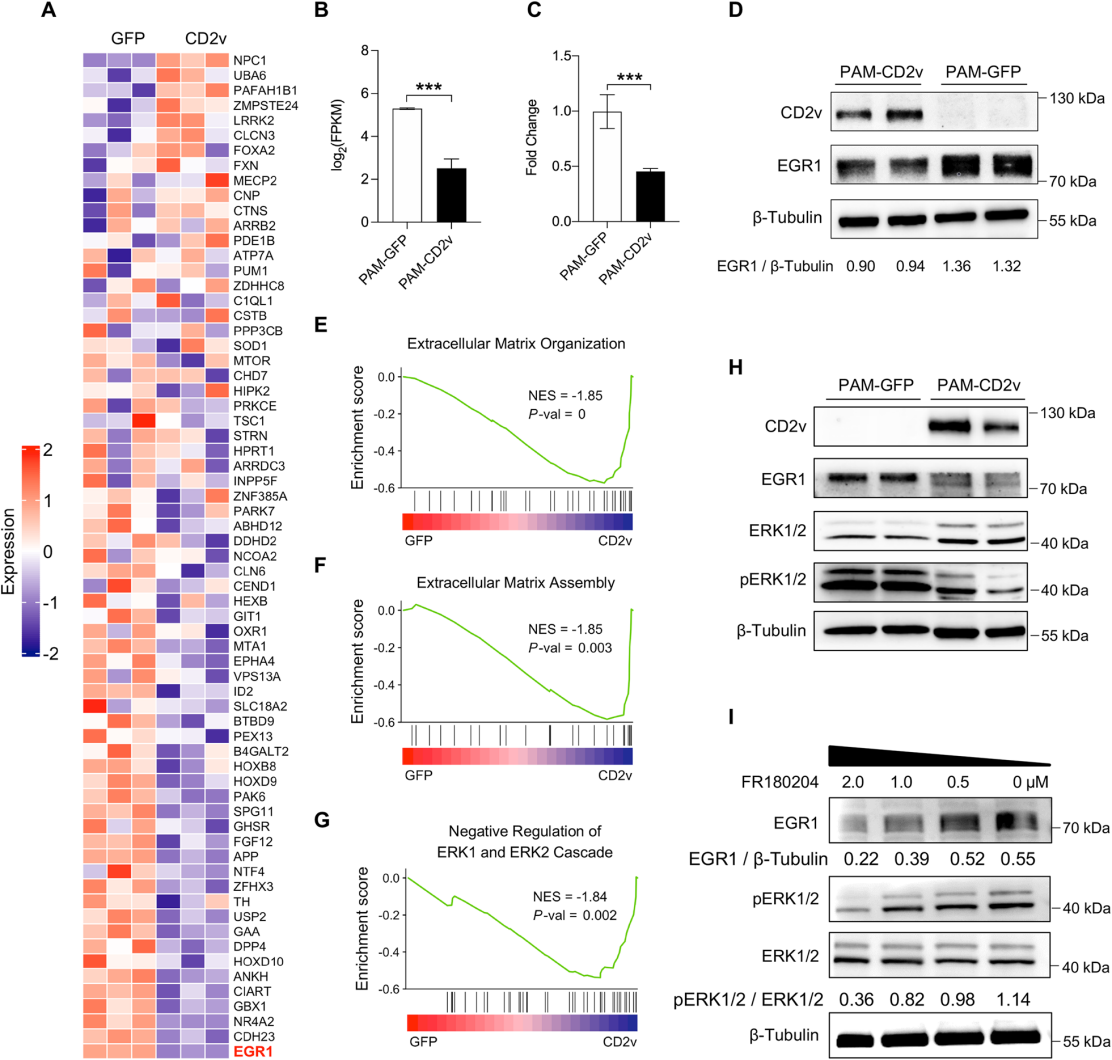
**CD2v downregulates EGR1 by inhibiting the activity of ERK1/2.** **A** Heatmap of all annotated swine genes in the locomotory behavior gene set. EGR1 is highlighted in red. **B** EGR1 expression in PAM-GFP and PAM-CD2v macrophages for RNA-seq (***, *P* < 0.001; *n* = 3). **C** EGR1 expression in PAM-GFP and PAM-CD2v macrophages was evaluated by RT-qPCR with specific primers (***, *P* < 0.001; *n* = 3). **D** EGR1 protein levels were determined by Western blotting in PAM-GFP and PAM-CD2v macrophages. **E** GSEA plots of extracellular matrix organization and extracellular matrix assembly gene sets. Rank ordered by log2Foldchange of PAM-CD2v versus PAM-GFP. **F** Representative Western blotting bands for CD2v, EGR1, ERK1/2, phosphorylation ERK1/2, and Tublin in PAM-GFP and PAM-GFP; **G** PAMs were treated with ERK1/2 inhibitor FR180204 or vehicle DMSO. The expression of EGR1 and phosphorylation ERK1/2 were evaluated by Western blotting.

Among all annotated genes that regulate the locomotory behavior of swine macrophages, EGR1 expression was ranked at the bottom of the list based on log_2_FoldChange (Figure 4A). In addition, of all DEGs in the RNA-seq results, EGR1 was one of the most significantly downregulated genes (Figure 2A and Figure 4B). We first validated the downregulation of EGR1 by RNA-seq using primers specific to EGR1 by RT-qPCR (Figure 4C). The protein levels of EGR1 were validated by Western blotting with a monoclonal antibody against EGR1. These results indicate that EGR1 was significantly downregulated in CD2v expressing PAMs (Figure 4D).

The above GSEA results indicated that CD2v negatively regulated extracellular matrix assembly and organization (Figures 4E and F), and the negative regulation of the ERK1 and ERK2 cascade (Figure 4G). Moreover, it has been established that the initiation of ERK1/2 signaling leads to the upregulation of EGR1 expression. We then investigated whether CD2v protein could inhibit ERK1/2 activity by downregulating EGR-1 expression. To this end, we analyzed the overall levels of ERK1/2 protein and phosphorylated ERK1/2 proteins in PAM-CD2v and PAM-GFP cells. Our findings indicated that phosphorylated ERK1/2 protein was reduced in PAM-CD2v cells as compared to PAM-GFP macrophages (Figure 4H). To further validate EGR1 downregulation due to the inhibition of ERK1/2, we treated swine PAMs with different doses of ERK1/2 inhibitor (FR180204) and examined total and phosphorylated EGR1/2 and EGR1 protein. The results demonstrated that both phosphorylated EGR1/2 and EGR1 decreased in proportion to the dosage (Figure 4I). Collectively, these outcomes suggest that CD2v downregulates EGR1 expression by inhibiting the ERK1/2 signaling pathway.

### EGR1 depleted in macrophage reduces cell migration

**Figure 5:**
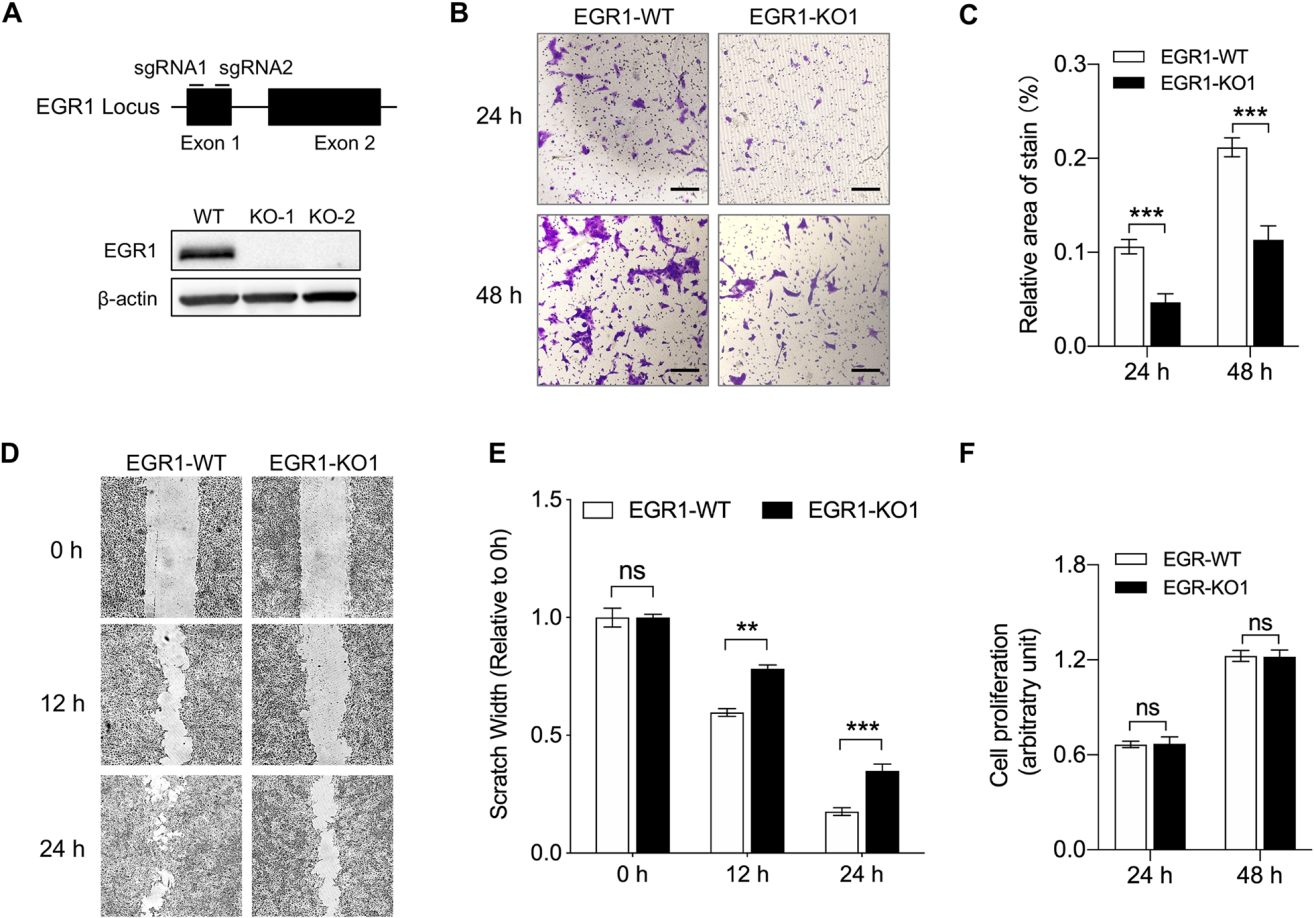
**EGR1 depleted in macrophage reduces cell migration**. **A** Schematic illustration of EGR gene locus of swine and small guide RNA location. The EGR1 expression was determined by Western blotting in EGR1-WT PAMs and EGR1-KO1 PAMs. **B** Representative images of EGR1-WT and EGR1-KO1 PAMs migration at 24 h and 48 h. Scale bar: 200 μm. **C** The number of migratory cells from the transwell assay was evaluated by five random fields’ relative staining areas (*** *P* < 0.001; *n* = 5). **D** Representative images of EGR1-WT and EGR1-KO1 PAMs at 12 and 24 h after initial scratch. **E** Quantification of scratch width EGR1-WT and EGR1-KO1 PAMs at different time points as indicated (** *P* < 0.01; *** *P* < 0.001; ns, not significant; *n* = 6). **F** Cell proliferation of EGR1-WT and EGR1-KO1 PAMs was determined by resazurin at different time points as indicated (ns, not significant; *n* = 8).

Previous studies have reported that EGR1 regulates the migration of different types of cells [50, 51]. We utilized CRISPR/Cas9 genome editing to create EGR1 knockout PAMs by targeting two locations within exon 1 of EGR1 and investigate the impact of EGR1 on swine macrophage migration (Figure 5A and Additional file 3). Vectors encoding Cas9 protein and small guide RNAs (sgRNAs) were transfected into PAMs. After collecting single clones, PCR was conducted using primers flanking the deleted region to confirm the absence of both alleles in the intervening deleted region. The EGR1 gene locus of EGR1 knockout (EGR1-KO) PAMs was confirmed through DNA sequencing (Additional file 3). The EGR1 protein was visualized via Western blotting in both EGR1-KO PAMs and parental PAMs, and the lack of EGR1 protein was verified in EGR1-KO PAM (Figure 5A).

The EGR1 knockout PAMs (EGR1-KO1) were used to perform in vitro assays to evaluate relative cell migration compared to control EGR1 wild-type (EGR1-WT) PAMs. The results from the transwell migration assay suggested decreased migration in EGR1-KO1 PAMs compared to that in control EGR1-WT PAMs, suggesting that EGR1 depletion inhibits macrophage migration (Figures 5B and C). EGR1 restriction of macrophage migration was also confirmed using wound healing assay (Figures 5D and E). In addition, the proliferation of EGR1-KO1 and EGR1-WT PAMs was evaluated by resazurin assay, and the results showed no impact of EGR1 knockout on macrophage proliferation (Figure 5F). We also performed a wound healing assay using another EGR1 knockout clone (EGR1-KO2). Consistent with the result from EGR1-KO1 PAMs, decreased migration in EGR1-KO2 PAMs compared to EGR1-WT PAMs was observed (Additional file 4).

### EGR1 overexpression in PAM-CD2v restores the ability of cell migration

**Figure 6:**
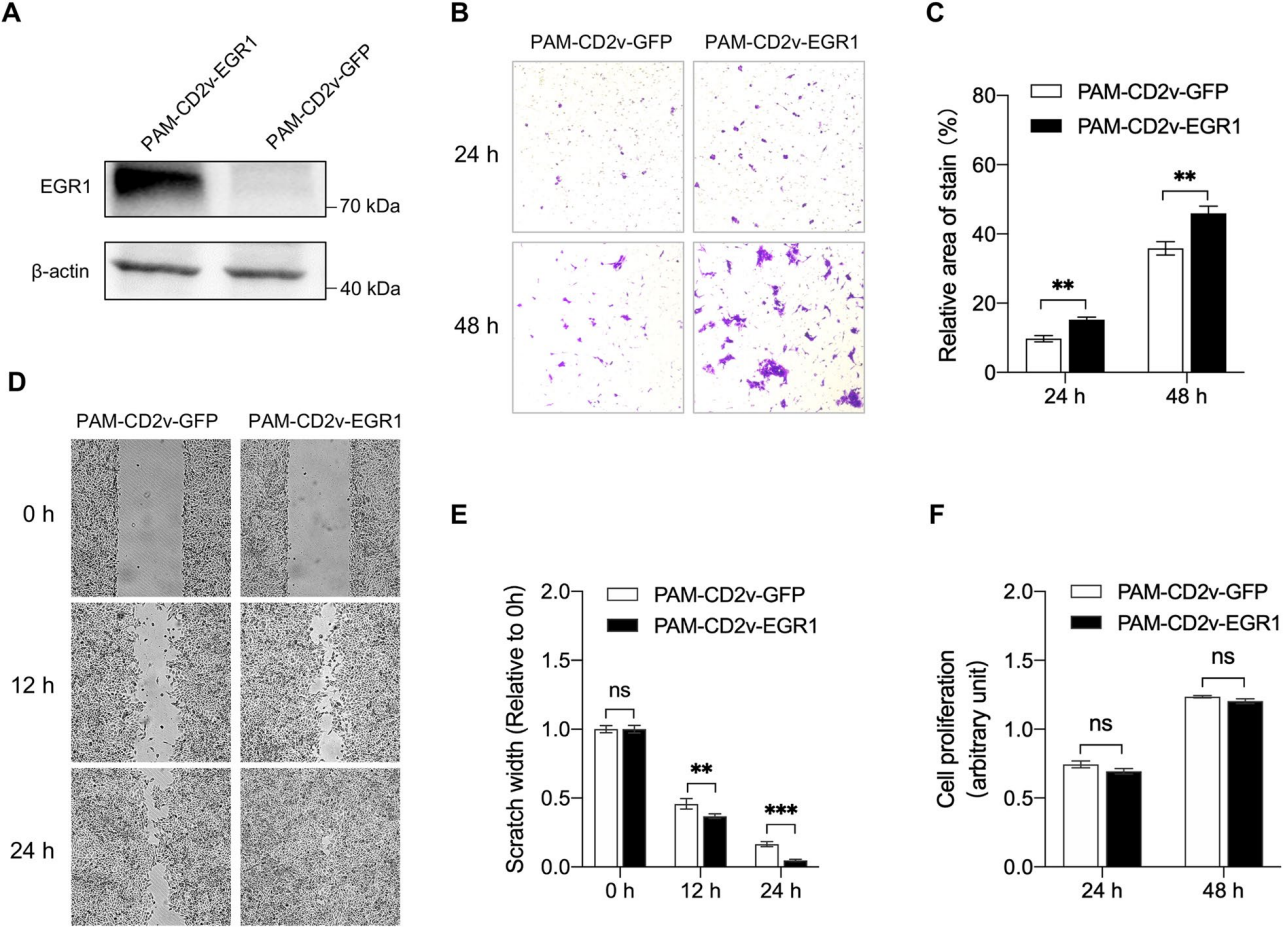
**EGR1 overexpression in PAM-CD2v restores the ability of cell migration**. **A** The EGR1 protein was determined by Western blotting in PAM-CD2v-EGR1 and PAM-CD2v-GFP cells. **B** Representative images of cell migration from PAM-CD2v-EGR1 and PAM-CD2v-GFP cells at 24 h and 48 h. **C** The number of migratory cells from the transwell assay was evaluated by five random fields’ relative staining areas (*** *P* < 0.001; *n* = 5). **D** Representative images of PAM-CD2v-EGR1 and PAM-CD2v-GFP cells at 12 and 24 h after initial scratch. **E** Quantification of scratch width of PAM-CD2v-EGR1 and PAM-CD2v-GFP cells at different time points as indicated (** *P* < 0.01; *** *P* < 0.001; ns, not significant; *n* = 6). **F** Cell proliferation of PAM-CD2v-EGR1 and PAM-CD2v-GFP were determined by resazurin at different time points as indicated (ns, not significant; *n* = 8).

To further investigate the role of EGR1 in CD2v-induced inhibition of macrophage migration, we stably overexpressed EGR1 or EGFP in PAM-CD2v cells using lentiviral vectors (designated PAM-CD2v-EGR1 and PAM-CD2v-GFP, respectively). EGR1 protein expression was confirmed in PAM-CD2v cells by Western blotting (Figure 6A). We then performed a transwell migration assay to evaluate PAM-CD2v-EGR1 cell migration compared to that of control GFP-overexpressing PAMs. The results showed increased migration of PAM-CD2v-EGR1 compared to the control PAM-CD2v-GFP, indicating that EGR1 overexpression restores the migration of swine macrophages (Figures 6B and C). The increased cell migration ability by EGR1 overexpression was also confirmed by a wound-healing assay (Figures 6D and E). The proliferation of EGR1-KO1 and EGR1-WT PAMs was evaluated by resazurin assay, and the results showed no impact of EGR1 overexpression on macrophage proliferation (Figure 6F). Altogether, these data indicate that CD2v reduced the migration of swine macrophages by downregulating EGR1 expression.

### EGR1 binds at active gene promoter regions of swine macrophage

EGR1 is a nuclear protein that functions as a transcriptional regulator. It belongs to the EGR family of C2H2-type zinc-finger proteins. The identification of direct targets of EGR1, with binding sites located within or near gene loci, would aid in comprehending the mechanism underlying EGR1-mediated regulation of swine macrophage migration. Therefore, we performed chromatin immunoprecipitation (ChIP) of EGR1 and H3K27 acetylation (H3K27ac), followed by deep sequencing in PAMs.

**Figure 7:**
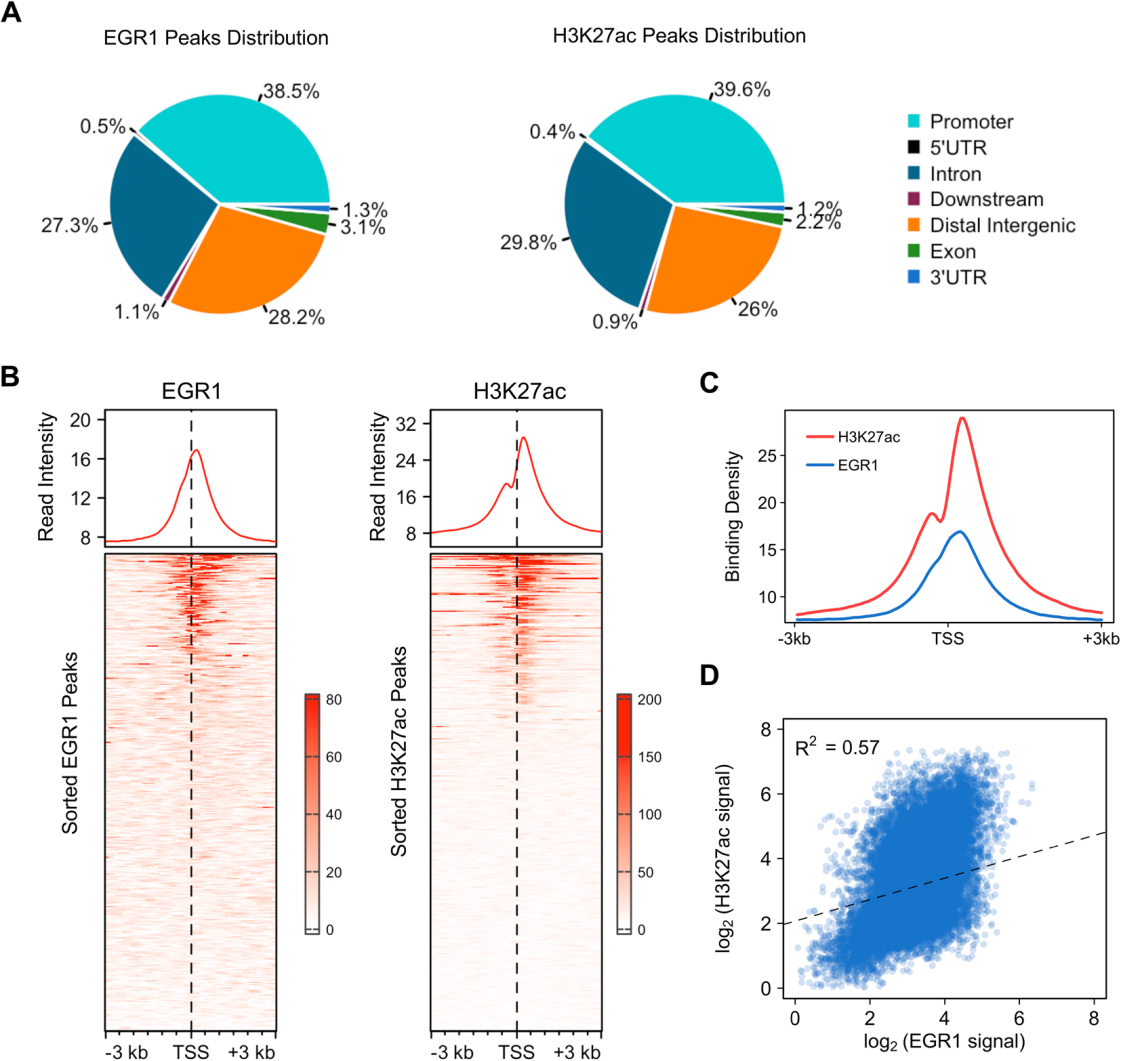
**ChIP-Seq analysis of genome-wide binding sites of EGR1 in PAMs**. **A** Distribution of EGR1-binding and H3K27ac enrichment sites in PAMs. Chromatin immunoprecipitation was conducted followed by deep sequencing (ChIP-seq) with the use of an EGR1 antibody and H3K27ac antibody. The total number of EGR1-bound and H3K27ac enrichment peaks was identified, and the percentage found within various genome regions was calculated. **B** Profile plots and heatmaps of the distribution of EGR1-binding and H3K27ac from ± 3 kb to genes’ transcriptional start site (TSS) are shown. The x-axis denotes the position from the TSS, and the y-axis shows the signal strength of 30 440 swine genes. **C** Overlayed profile plot of EGR1 and H3K27ac ChIP-seq read distribution within 3 kb of TSS. **D** Quantitative correlation of signal strength of EGR1 binding and H3K27ac enrichment at all genes TSSs. The linear regression trendline and the correlation coefficient-square are displayed.

ChIP-seq analysis revealed the presence of 25 274 binding peaks for EGR1 protein. The majority of EGR1 binding sites (about two-thirds) of EGR1 were situated in intronic and distal intergenic regions (Figure 7A and Additional file 8), indicating that EGR1 may potentially regulate gene expression by binding to these distal regulatory elements [52, 53]. Around 38.5% of the binding sites were located at or near promoter regions (Figure 7A and B), representing 8793 genes out of 30 440 genes. Additional analysis of the distance between the closest transcription start sites (TSS) and peaks indicated that 86% of EGR1 peaks were located downstream or 1 kb upstream of the nearest TSS (Additional file 5). This suggests that EGR is recruited to these regions of the genome in swine macrophages to directly modulate the transcriptional activity of neighboring genes.

In the parallel experiment, a total of 28 806 binding peaks were observed through H3K27ac immunoprecipitation. It was observed that 39.6% of the enrichment sites were located at or near the promoter regions (Figures 7A and B, and Additional file 9). Among these enrichment sites, 88.2% were found within 1 kb upstream or downstream of the nearest TSS (Additional file 5). Signal intensity for EGR1 and H3K27ac binding around TSS regions was visualized, revealing that EGR1 binding peaks in swine macrophages overlapped with the bimodal distribution of the histone mark H3K27ac (Figure 7C). Scatter plot analysis showed that significant correlation between EGR1 occupancy and H3K27as enrichment on all TSS in the swine macrophage genome (Figure 7D). These results demonstrated that EGR1 binds to the promoter region of active genes in swine macrophages.

### EGR1 binds at the promoter regions of cell locomotion-related genes

**Figure 8:**
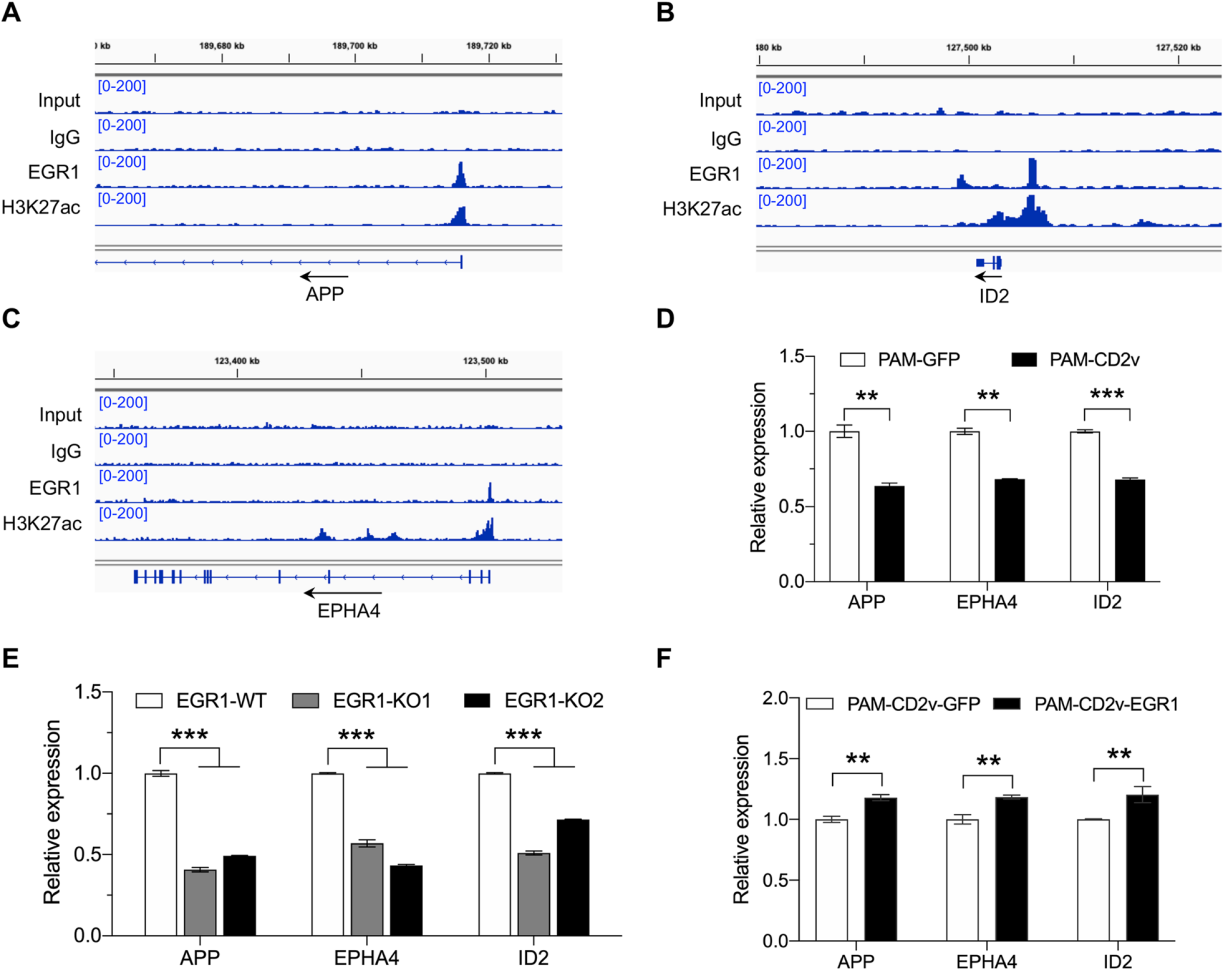
**EGR1 binds at the promoter regions of cell locomotion-related genes**. Screenshot from IGV showing DNA sequence tag densities at gene APP (**A**), ID2 (**B**), and EPHA4 (**C**) locus following ChIP-seq with the indicated antibodies in PAMs. The maximum coverage for each track is shown on the left. **D** Relative expression levels of gene APP, EPHA4, and ID2 in PAM-GFP and PAM-CD2v (** *P* < 0.01; *** *P* < 0.001; *n* = 3). **E** Relative expression levels of gene APP, EPHA4, and ID2 in EGR1-WT and EGR1-KO PAMs (*** *P* < 0.001; *n* = 3). (F) Relative expression levels of gene APP, EPHA4, and ID2 in PAM-CD2v-EGR1 and PAM-CD2v-GFP (** *P* < 0.01; *n* = 3).

To address how EGR1 influences swine macrophage migration, we visualized EGR1 binding sites and H3K27ac enrichment sites around locomotory behavior-related genes. Among these genes, APP [54, 55], ID2 [56], and EPHA4 [57] have been demonstrated to facilitate cell migration in various types of cells. As shown in Figures 8A, B and C, respectively, EGR1 could bind to the upstream TSS of these three genes with the histone mark H3K27ac. To assess the impact of EGR1 on the expression of the aforementioned three genes, we quantified the expression levels of these genes in PAM-CD2v, PAM-GFP, EGR1-KO, EGR1-WT macrophages, PAM-CD2v-EGR1, and PAM-CD2v-GFP cells. The results showed that the expression levels of APP, ID2, and EPHA4 were significantly reduced in PAM-CD2v compared to the control group PAM-GFP (Figure 8D). Similarly, EGR1-KO PAMs showed significantly lower levels of APP, ID2, and EPHA4 expression compared to EGR1-WT PAMs (Figure 8E). However, APP, ID2, and EPHA4 gene expression levels were restored in PAM-CD2v-EGR1 cells compared to those in control PAM-CD2v-GFP cells (Figure 8F). Taken together, these findings suggest that CD2v reduces the expression of locomotor genes by downregulating EGR1.

### CD2v dampens the response to the ERK1/2 pathway by downregulating the expression of EGR1

**Figure 9:**
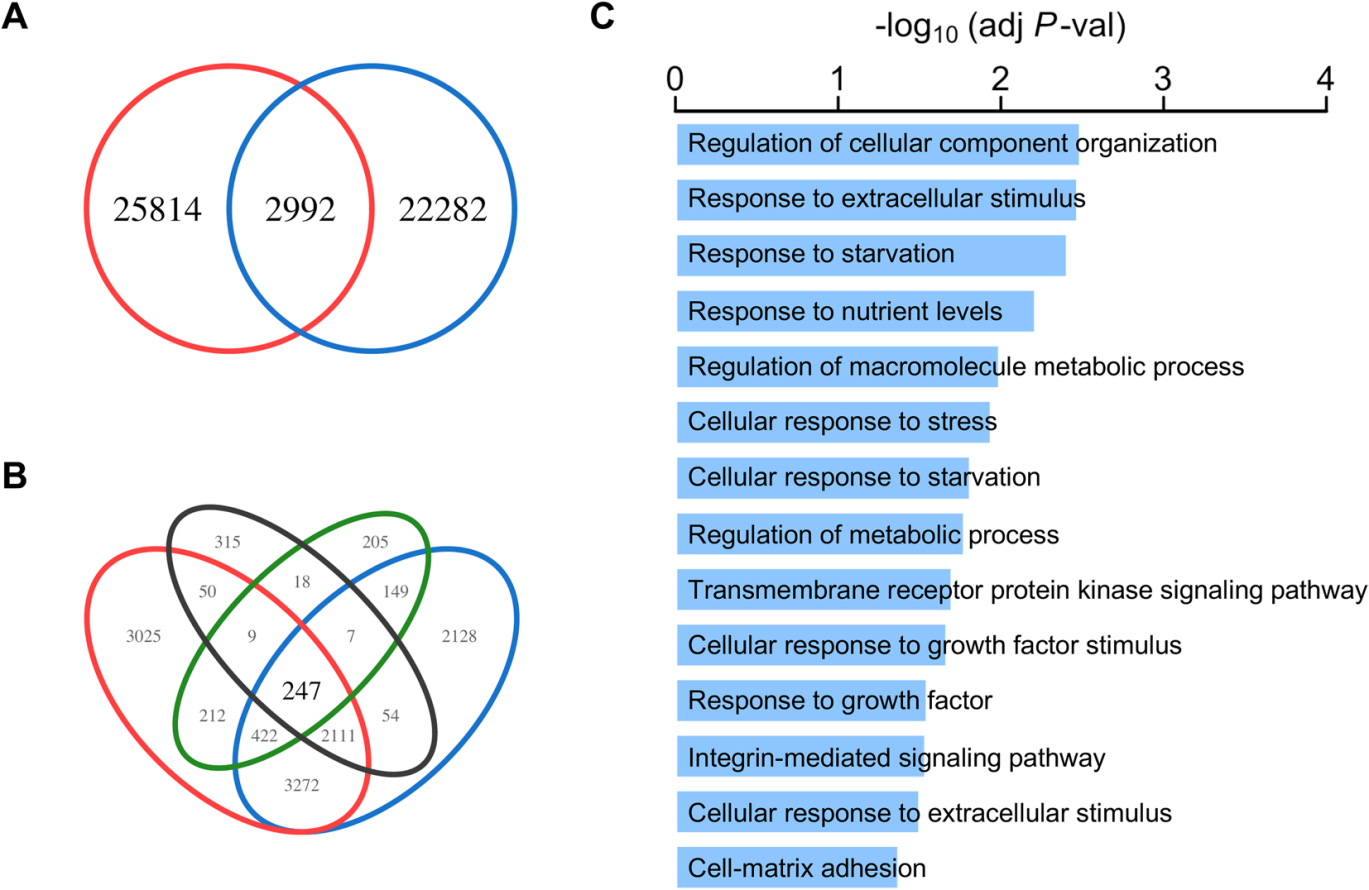
**CD2v dampens the response to the ERK1/2 pathway through EGR1.** **A** The Venn diagram illustrates the overlapping of EGR1 (shown in blue) and H3K27ac (shown in red) binding peaks. **B** The Venn diagram demonstrates the overlapped genes that are co-occupied by EGR1 and H3K27ac (black), genes containing EGR1 binding peaks within 1 kb up or downstream of their TSS (red), genes containing H3K27ac binding peaks within 1 kb up or downstream of their TSS (blue), and differential expressed genes (adjust *p* < 0.05 and log_2_FoldChange < 0) in PAM-CD2v versus PAM-GFP (green). **C** GO term enrichment analysis of 247 overlapped genes from B.

A variety of stimuli, such as cytokines, growth factors, mitogens, and stress, can induce the EGR1 expression via different MAPK pathways, including the ERK signaling pathway. We showed that CD2v downregulated EGR1 by inhibiting ERK1/2 activity. To further confirm the function of CD2v in regulating the ERK1/2 signaling pathway, we evaluated the downregulated genes in PAM-CD2v and associated them with EGR1 binding peaks in the ChIP-seq data. To do so, we first intersected the EGR1 binding peaks with H3K27ac enrichment peaks to find the genes co-occupied with EGR1 and H3K27ac binding. As shown in Figure 9A, there were 2992 sites located within or near the 2811 gene loci (Additional file 10). We then intersected these genes, genes that contained EGR1 binding peaks and H3K27ac binding peaks within 1 kb downstream or upstream of their TSS, and the differentially expressed genes (adjusted *P* < 0.05 and log_2_FoldChange < 0) in PAM-CD2v compared to PAM-GFP. A total of 247 overlapping genes were identified (Figure 9B). Gene ontology analysis of these overlapping genes identified the most significantly enriched terms involved in extracellular stimuli, such as stress, growth factor, and starvation (Figure 9C). This finding further confirmed that CD2v expression in swine macrophages inhibited the response to the ERK1/2 pathway through the downregulation of EGR1 expression.

### CD2v inhibits the expression of inflammatory cytokines in swine macrophage

**Figure. 10:**
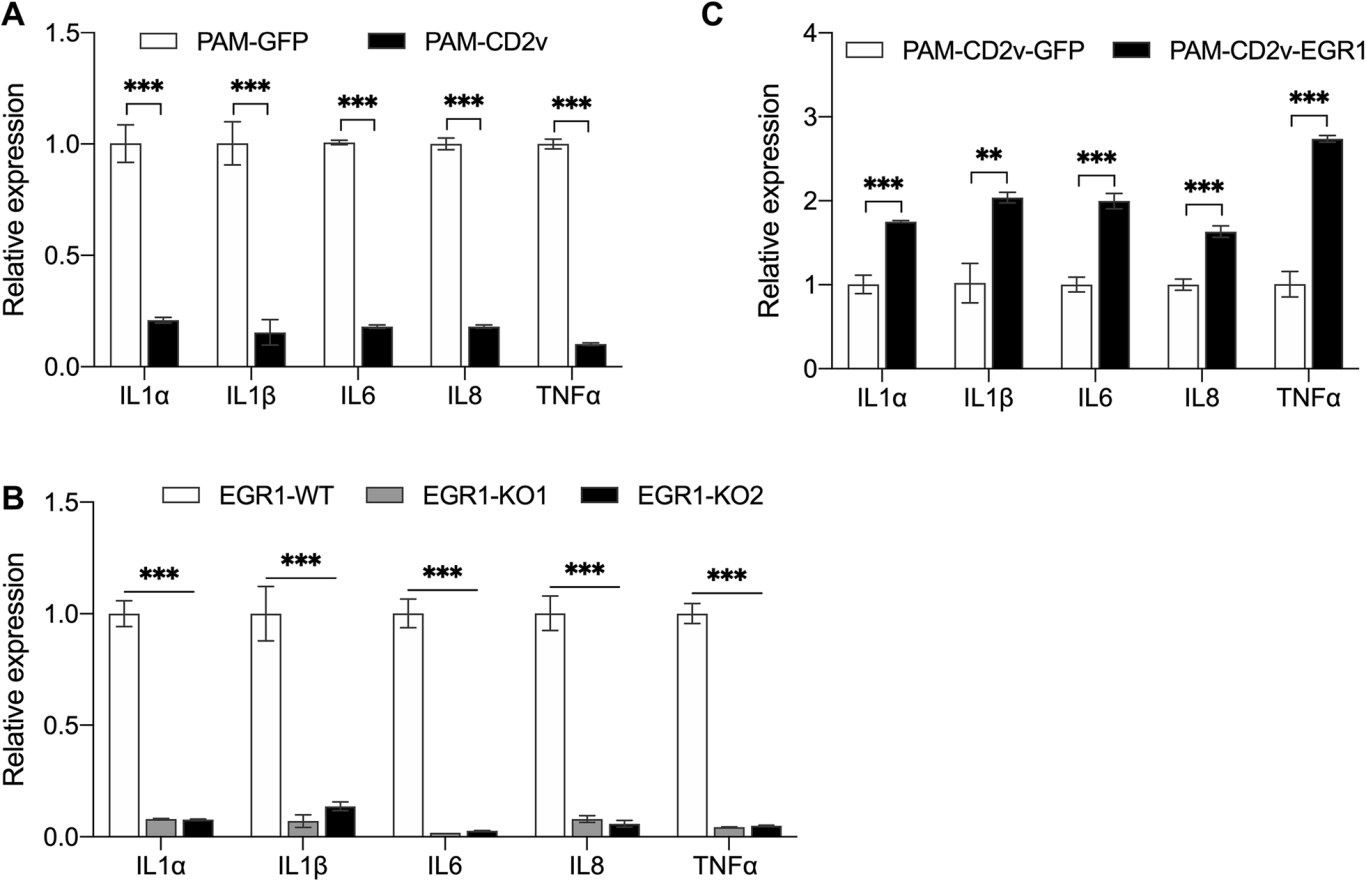
**CD2v inhibits the expression of inflammatory cytokines in swine macrophages.** Various derivatives of PAM were treated with 2 μg/mL LPS for 4 h. The expression levels of inflammatory cytokine genes were evaluated by qPCR. **A** Relative expression levels of gene IL-1α, IL-1β, IL6, IL8, and TNFα in PAM-GFP and PAM-CD2v (*** *P* < 0.001; *n* = 4). **B** Relative expression levels of gene IL-1α, IL-1β, IL6, IL8, and TNFα in EGR1-WT and EGR1-KO PAMs (** *P* < 0.01; *** *P* < 0.001; *n* = 4). **C** Relative expression levels of gene IL-1α, IL-1β, IL6, IL8, and TNFα in PAM-CD2v-EGR1 and PAM-CD2v-GFP (** *P* < 0.01; *** *P* < 0.001; *n* = 4).

EGR1 can induce the expression of several chemokine/cytokine genes, including TNFα, IL6, and IL8 [28–30, 58], which play essential roles in regulating inflammation and immune responses. We explored whether CD2v regulates the expression of these genes through EGR1 following an external stimulus. PAM-CD2v, PAM-GFP, EGR1-WT PAM, and EGR1-KO PAM (two EGR1 knockout clones, EGR1-KO1 and EGR1-KO2) PAM-CD2v-EGR1 and PAM-CD2v-GFP, were treated with LPS for 4 h. Cytokine expression (IL1α, IL1β, IL6, IL8, and TNFα) was measured by RT-qPCR. These results demonstrated that CD2v expression in PAMs led to a significant reduction in the expression of all five cytokines when compared to the control PAM-GFP (Figure 10A). Reduced expression of these cytokines was also observed in the EGR1-KO PAMs (Figure 10B). However, the overexpression of EGR1 in PAM-CD2v significantly increased the expression of all five cytokines compared to the control PAM-CD2v-GFP (Figure 10C). These results indicate that CD2v inhibited inflammatory cytokine expression in swine macrophages by downregulating EGR1 expression.

## Discussion

ASFV CD2v shares structural similarities with the host CD2 protein. The host CD2 molecule is a cell surface glycoprotein that plays a crucial role in the immune response. It is primarily expressed in T cells, although it can also be found in natural killer (NK) cells and some subsets of B cells. The main function of CD2 is to mediate cell adhesion and signaling. CD2 interacts with its ligand, CD58 (also known as LFA-3), which is expressed on antigen-presenting cells (APCs) such as dendritic cells, macrophages, and B cells. This interaction between CD2 and CD58 is important for the formation of stable T cell-APC conjugates. CD2-CD58 binding facilitates the adhesion and conjugation of T cells to APCs, allowing for efficient antigen recognition and presentation. This interaction is crucial for T cell activation, as it provides a stable platform for the formation of the immunological synapse, a specialized structure that facilitates communication between T cells and APCs. In addition to cell adhesion, CD2 signaling is involved in various cellular processes. Upon binding to CD58, CD2 triggers intracellular signaling cascades that lead to T cell activation, proliferation, and cytokine production. CD2 signaling also contributes to the regulation of T cell differentiation and survival. Furthermore, CD2 is involved in the regulation of T cell migration. It interacts with other molecules on the surface of endothelial cells, such as CD48 and CD59, facilitating T cell extravasation from the bloodstream into tissues during an immune response extracellular and intracellular signaling events [59–61]. CD2v is expressed on the surface of macrophages infected with ASFV [6] and is necessary for the virus-induced rosetting of erythrocytes around infected cells. In this study, a stable CD2v-expressing swine macrophage cell line was generated. Consistent with a previous report [24], CD2v was observed in the cell membrane and perinuclear region. CD2v-expressing macrophages had hemadsorption ability.

Almost all cell types can migrate, but this is essential for immune cells to respond properly. Here, RNA-seq of PAM-CD2v and PAM-GFP was performed to determine the biological processes regulated by ASFV CD2v in swine macrophages. GO analysis indicated that CD2v affects the extracellular matrix organization and assembly. GSEA indicated that CD2v negatively regulated the locomotor behavior of swine macrophages. Our wound healing and transwell assays confirmed that CD2v inhibits swine macrophage migration. ASFV primarily infects mononuclear phagocytic cells, such as macrophages, monocytes, and dendritic cells. These cells are crucial in inducing innate immune responses and presenting antigens to T cells to activate an adaptive immune response [3–5]. The migration of these cells is essential for immune surveillance and response initiation. Our findings suggest that reduced migration of these cells could impair the induction of both innate and adaptive immune responses.

Infection of cultured peripheral blood mononuclear cells with ASFV results in a decrease in mitogen-dependent proliferation of uninfected lymphocytes. The elimination of this effect is observed upon deletion of the CD2v gene [12]. This finding indicates that CD2v may inhibit the activity of MAPK, which is essential for phosphorylation of ERK1/2. The ERK1/2 MAP kinase pathway plays a crucial role in transmitting extracellular signals to the nucleus, thereby regulating cell proliferation, cell cycle, and cell development [62]. It is well known that ERK1/2 is involved in the Ras-Raf-MEK-ERK signal transduction pathway, which regulates a wide range of processes such as cell migration, metabolism, differentiation, and transcription [63]. In normal cells, continuous activation of ERK1/2 is essential for the progression from G1 to S phase of the cell cycle and is related to the activation of positive regulators of the cell cycle and deactivation of antiproliferative genes [64]. In this study, we found that CD2v inhibited the phosphorylation of ERK1/2. It has been reported that CD2v is processed and released from infected macrophages or CD2v-expressing cells [24, 49, 65]. The released soluble CD2v may interact with or invade lymphocytes and affect the biological processes of these lymphocytes. A recent study showed that a C-terminal 88 amino acid fragment from CD2v can enter cells [66]. Our findings may explain why CD2v reduces the mitogen-dependent proliferation of lymphocytes. However, the underlying mechanism of how CD2v inhibits the activity of ERK1/2 needs to be further investigated.

EGR1 plays a crucial role in the growth, maintenance, and repair of connective tissues, primarily by controlling the extracellular matrix [67]. GSEA from RNA-seq showed that CD2v affected the extracellular matrix organization and assembly. Conceivably, CD2v exerts its effects by downregulating EGR1. Different stimuli like cytokines, growth factors, mitogens, and stress activate the expression of EGR1 via distinct MAPK pathways including the extracellular signal-regulated kinase [26, 27]. We found that EGR1 was significantly downregulated in CD2v expressing swine macrophages by dampening the activity of ERK1/2. Previous studies have shown that EGR1 regulates the migration of different cell types [50, 51]. We confirmed that EGR1 depletion in swine macrophages restricts cell migration. We further defined the cistrome of EGR1 in swine macrophages by using ChIP-seq. We showed that EGR1 and H3K27ac co-occupied the gene promoter regions in swine macrophages, including the promoter regions of cell locomotion-related genes.

EGR1 governs inflammation in diverse tissues and promotes inflammation in various animal models [31, 68, 69]. We showed that suppression of EGR1 by CD2v resulted in the suppression of inflammatory gene expression, including IL1α, IL1β, IL6, IL8, and TNFα, which are critical for inflammation and the immune response. IL-1 is predominantly linked with innate immunity and has a significant function in acquired immunity [70]. IL-6 is a pleiotropic cytokine that is synthesized in response to infections and tissue damage [71]. It aids in host defense by inducing acute-phase responses and immune reactions. IL-8 acts as a chemotactic factor that attracts neutrophils, basophils, and T cells during inflammation. Tumor necrosis factor (TNF)α is a potent paracrine and endocrine agent that mediates inflammatory and immune processes. TNFα and IL6 have been shown to enhance macrophage activation and antigen presentation and to regulate immunity through various mechanisms [72]. Interestingly, our study found that the expression of these cytokines was dramatically decreased by CD2v expression in swine macrophages. This suggests that CD2v may play a significant role in modulating immune responses by regulating the expression of key inflammatory genes.

This study demonstrated that the CD2v protein of ASFV inhibits the migration of swine macrophages and the expression of inflammatory cytokines by downregulating the expression of the transcription factor EGR1 through dampening the activity of ERK1/2 using an immortalized porcine alveolar macrophage line (iPAM, 3D4/21). Although the iPAM (3D4/21) cell line was extensively used to study ASFV [73–77], they likely do not completely replicate all aspects of infection in primary cells, such as porcine alveolar macrophages. It would have been beneficial to investigate the effects of CD2v-deleted ASFV on macrophage migration and the expression of inflammatory cytokines. However, it is important to note that during viral infections, the role of CD2v in cell migration may become redundant. This is because infected macrophages may already experience a reduction in their migration capabilities, even without the presence of CD2v. ASFV encodes more than 150 proteins. In addition to CD2v, there might be other factors that can inhibit the expression level of EGR1. These factors could also play a significant role in regulating the cellular response and ultimately affect cell migration and the expression of inflammatory cytokines. We conducted gene expression analysis on RNA-seq data obtained from another research group (Gene Expression Omnibus: GSE145954) [78]. Our findings indicate that ASFV significantly reduces the expression level of EGR1 (Additional file 12). Consequently, further investigations are required to elucidate the additional factors that play a critical role in the intricate network of pathways involved in the regulation of EGR1 expression. These studies will also contribute to a more comprehensive understanding of the role of CD2v in ASFV pathogenesis.

In summary, the findings of our study reveal that CD2v of ASFV impairs swine macrophage migration by modulating the expression of the transcription factor EGR1 through the inhibition of ERK1/2 activity. We observed that EGR1 and H3K27ac co-occupy the promoter regions of genes involved in cell locomotion in swine macrophages, suggesting that CD2v may influence cell movement by targeting this axis. In addition, our results demonstrate that CD2v downregulates the expression of inflammatory cytokines via EGR1-mediated signaling in swine macrophages. These novel findings highlight the immunomodulatory potential of CD2v in ASFV pathogenesis.

### Supplementary Information

**Additional file 1: Schematic illustration of lentiviral vectors used for ASFV CD2v overexpression in PAMs. The GFP vector was constructed as a control.**

**Additional file 2:  Overview of RNA-seq results**. (A) Here are the numbers of total and uniquely aligned reads for the PAM-GFP and PAM-CD2v cell lines. (B) A correlation heat map of PAM-GFP and PAM-CD2v is presented, showing the relationship between their gene expression. The colors in the heat map indicate pairwise Pearson correlations, which are calculated using the expression values of all genes. The hierarchical clustering is based on the negative correlation distance. (C) PCA analysis was conducted on the FPKM expression matrix of all samples.

**Additional file 3:  Generating EGR1 knockout cell lines**. (A) Schematic illustration of EGR gene locus of swine, small guide RNA sequences location (blue line), primer (red arrow) flanking deletion region. (B) PAMs were transfected with sgRNA1 and sgRNA2 plus Cas9. 24 h after transfection, transfected cells were subjected to puromycin selection for 48 h. Subsequently, the cells that were resistant to puromycin were seeded into 96-well plates using a dilution method that limited their density. Each clone was carefully chosen and allowed to grow and expand. PCR was carried out to amplify the region inside of two sgRNAs. Agarose gel image of PCR products that amplify EGR1 sgRNAs targeting regions. The red asterisk indicated that both alleles contained deletions. Predicted PCR product size with deletion: 441 bp; Predicted PCR product size without deletion: 574 bp. (C) DNA sequencing result from the EGR1-KO1 clone was presented. sgRNA sequences in blue; Protospacer adjacent motif (PAM) sequence in red; black triangles show the predicted cleavage sites.

**Additional file 4:  Representative images of EGR1-WT and EGR1-KO2 PAMs at 12 and 24 h after initial scratch**.

**Additional file 5:  Distribution of EGR1 and H3K27ac peaks at gene promoters**. (A) EGR1 peaks distribution at nearest gene promoter; (B) H3K27ac peaks distribution at nearest gene promoter.

**Additional file 6:  The output of differentially expressed genes between PAM-CD2v and PAM-GFP from DESeq2 software**.

**Additional file 7:  Gene sets were negatively regulated by CD2v from gene sets enrichment analysis**.

**Additional file 8:  EGR1 binding peaks Annotation in swine macrophage from ChIP-seq**.

**Additional file 9:  H3K27ac binding peaks Annotation in swine macrophage from ChIP-seq**.

**Additional file 10:  EGR1 and H3K27ac differential binding peaks in swine macrophage from ChIP-seq**.

**Additional file 11:  Oligonucleotide sequences used in this study**.

**Additional file 12:  EGR1 expression in ASFV-infected and MOCK-infected primary swine macrophage**.

## Data Availability

RNA-seq and ChIP-seq data were deposited in the Gene Expression Omnibus database, with accession codes GSE189708 and GSE189087. All other data are available from the authors upon request.
