# Supplementary figures and images for: The CD2v protein of African swine fever virus inhibits macrophage migration and inflammatory cytokines expression by downregulating EGR1 expression through dampening ERK1/2 activity
Source: Vet Res. 2023 Nov 15;54:106. doi: 10.1186/s13567-023-01239-w (PMC10648359; doi:10.1186/s13567-023-01239-w)

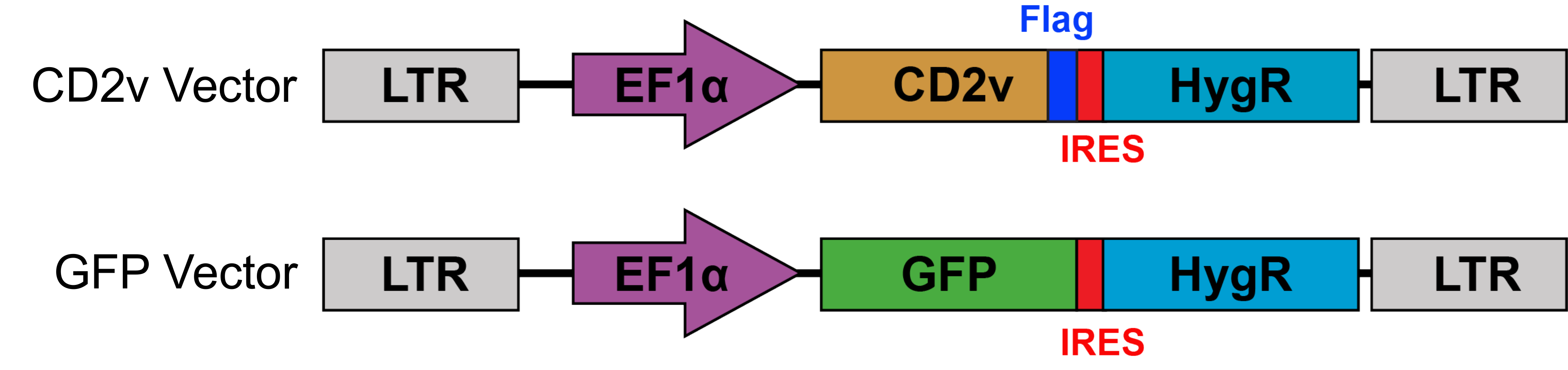

Supplement: Supplementary file 1 — Additional file 1: Schematic illustration of lentiviral vectors used for ASFV CD2v overexpression in PAMs. The GFP vector was constructed as a control. [file 13567_2023_1239_MOESM1_ESM.tif]

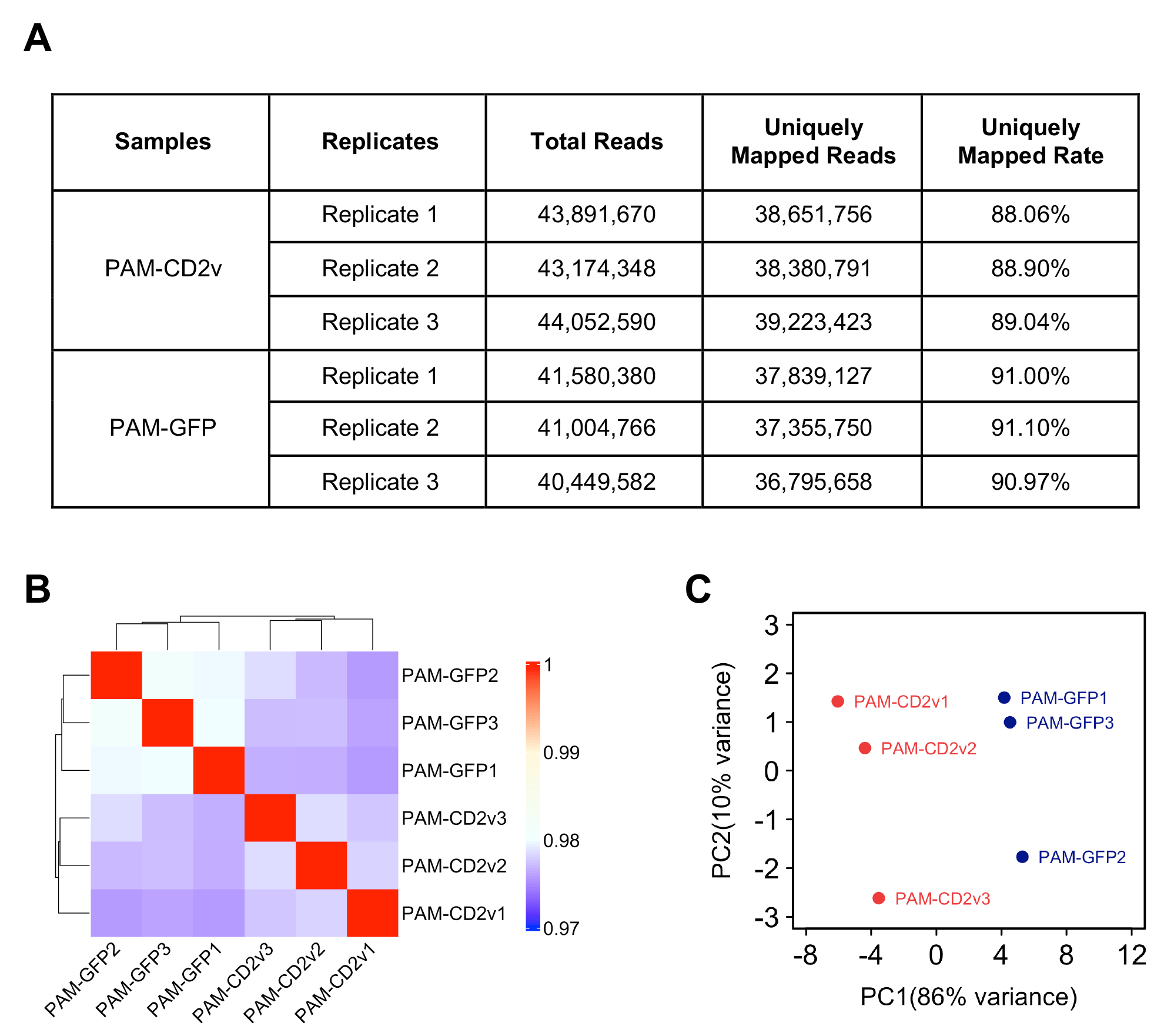

Supplement: Supplementary file 2 — Additional file 2: Overview of RNA-seq results. (A) Here are the numbers of total and uniquely aligned reads for the PAM-GFP and PAM-CD2v cell lines. (B) A correlation heat map of PAM-GFP and PAM-CD2v is presented, showing the relationship between their gene expression. The colors in the heat map indicate pairwise Pearson correlations, which are calculated using the expression values of all genes. The hierarchical clustering is based on the negative correlation distance. (C) PCA analysis was conducted on the FPKM expression matrix of all samples. [file 13567_2023_1239_MOESM2_ESM.tif]

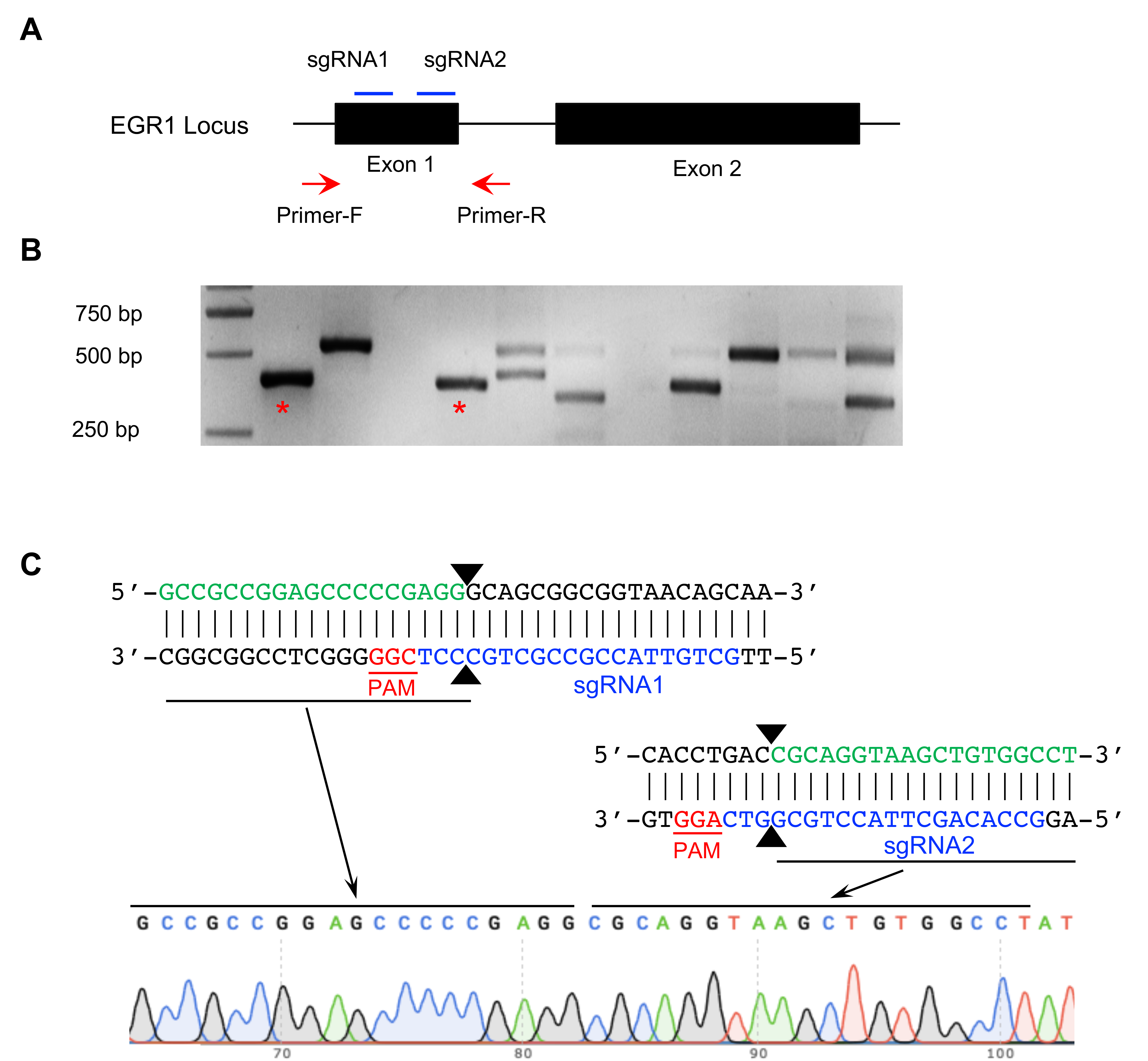

Supplement: Supplementary file 3 — Additional file 3: Generating EGR1 knockout cell lines. (A) Schematic illustration of EGR gene locus of swine, small guide RNA sequences location (blue line), primer (red arrow) flanking deletion region. (B) PAMs were transfected with sgRNA1 and sgRNA2 plus Cas9. 24 h after transfection, transfected cells were subjected to puromycin selection for 48 h. Subsequently, the cells that were resistant to puromycin were seeded into 96-well plates using a dilution method that limited their density. Each clone was carefully chosen and allowed to grow and expand. PCR was carried out to amplify the region inside of two sgRNAs. Agarose gel image of PCR products that amplify EGR1 sgRNAs targeting regions. The red asterisk indicated that both alleles contained deletions. Predicted PCR product size with deletion: 441 bp; Predicted PCR product size without deletion: 574 bp. (C) DNA sequencing result from the EGR1-KO1 clone was presented. sgRNA sequences in blue; Protospacer adjacent motif (PAM) sequence in red; black triangles show the predicted cleavage sites. [file 13567_2023_1239_MOESM3_ESM.tif]

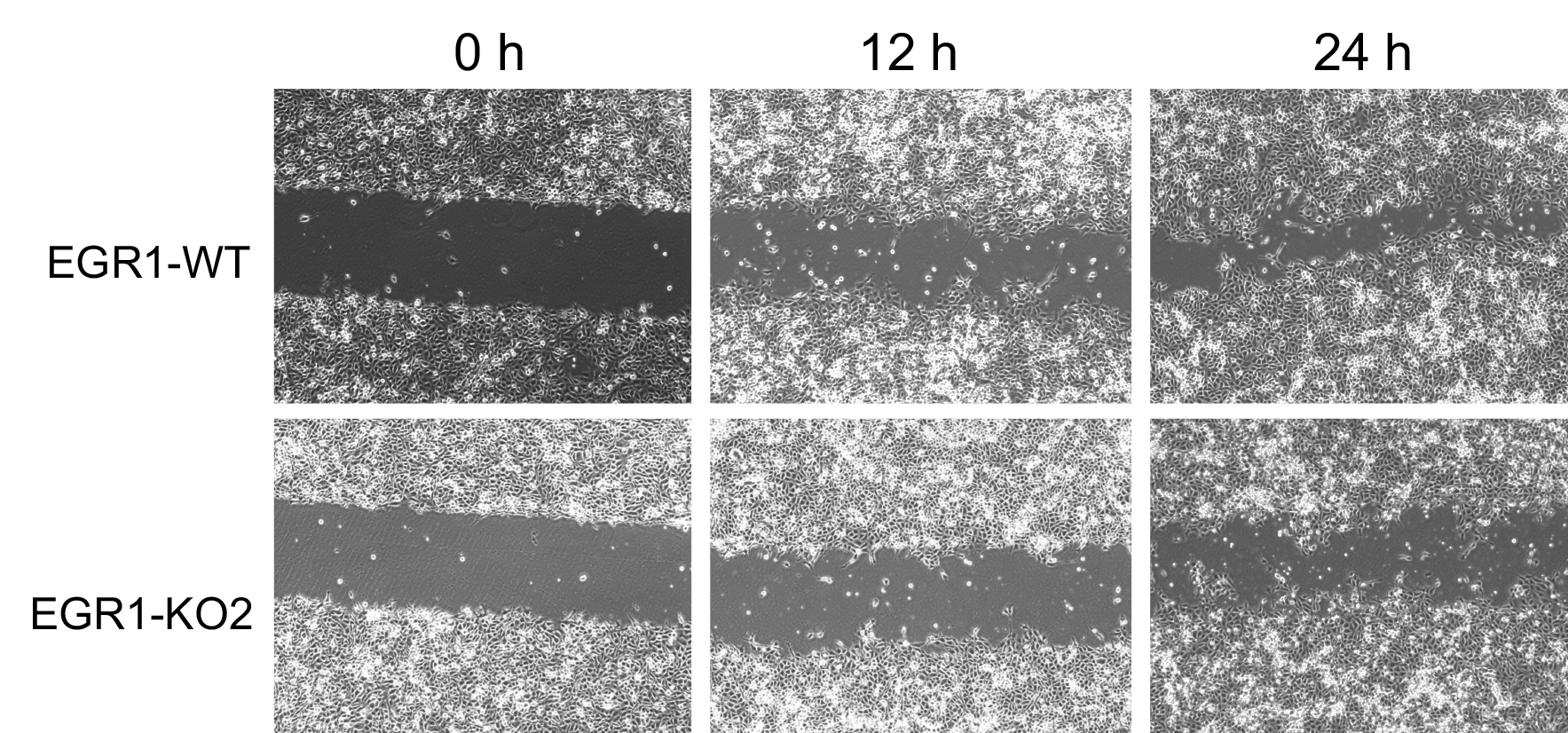

Supplement: Supplementary file 4 — Additional file 4: Representative images of EGR1-WT and EGR1-KO2 PAMs at 12 and 24 h after initial scratch. [file 13567_2023_1239_MOESM4_ESM.tif]

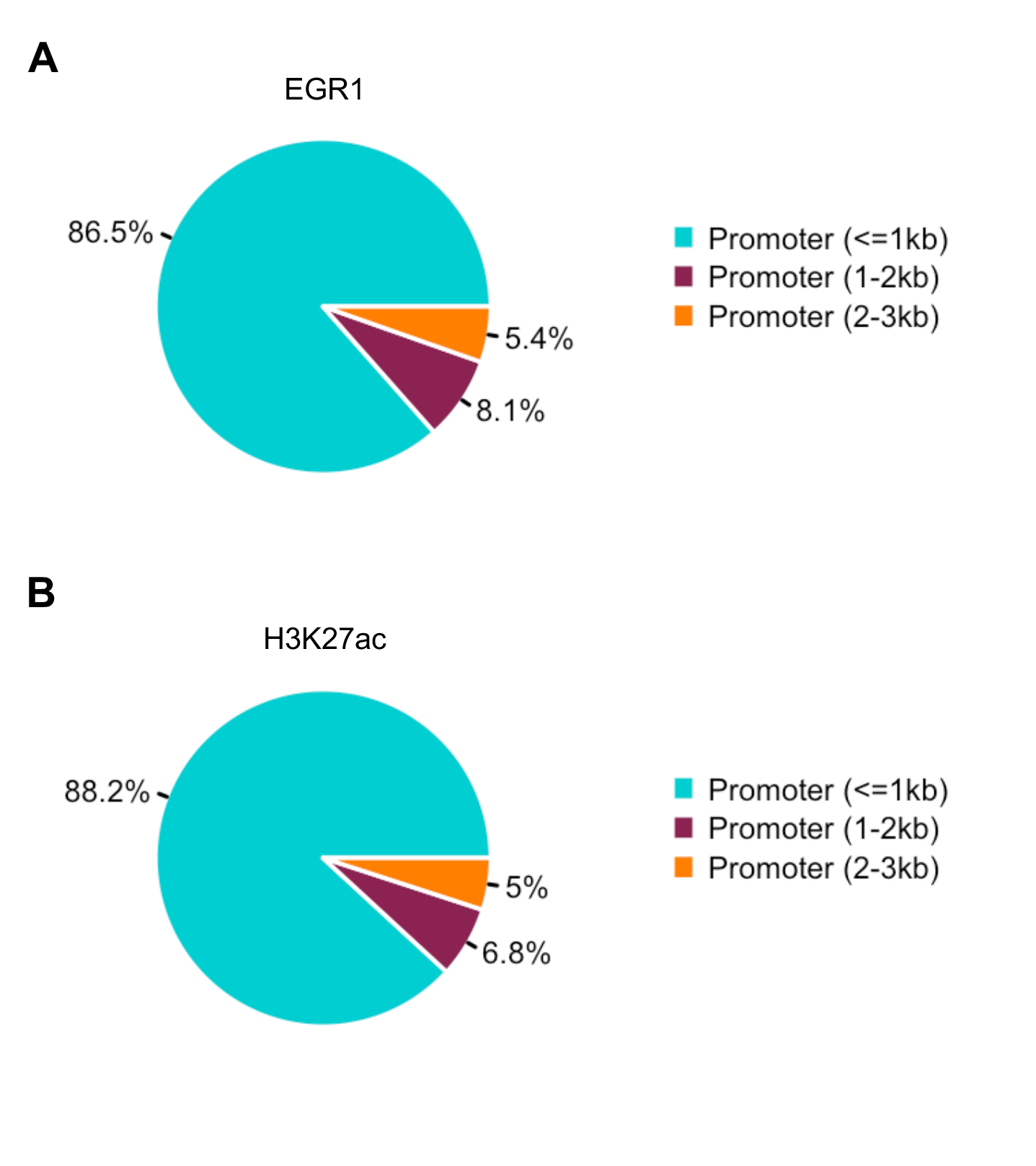

Supplement: Supplementary file 5 — Additional file 5: Distribution of EGR1 and H3K27ac peaks at gene promoters. (A) EGR1 peaks distribution at nearest gene promoter; (B) H3K27ac peaks distribution at nearest gene promoter. [file 13567_2023_1239_MOESM5_ESM.tif]

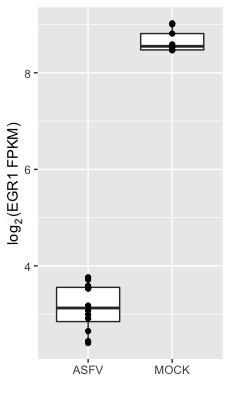

Supplement: Supplementary file 12 — Additional file 12: EGR1 expression in ASFV-infected and MOCK-infected primary swine macrophage. [file 13567_2023_1239_MOESM12_ESM.tif]
