# Supplementary material for: The CD2v protein of African swine fever virus inhibits macrophage migration and inflammatory cytokines expression by downregulating EGR1 expression through dampening ERK1/2 activity
Source: Vet Res. 2023 Nov 15;54:106. doi: 10.1186/s13567-023-01239-w (PMC10648359; doi:10.1186/s13567-023-01239-w)
Supplement: Supplementary file 11 — Additional file 11: Oligonucleotide sequences used in this study. [file 13567_2023_1239_MOESM11_ESM.docx]

| **Oligo name** | **Sequence (5’-3’)** | **Purpose** |
| --- | --- | --- |
| TNFα-F | TGCCTCAGCCTCTTCTCCTTCC | qPCR |
| TNFα-R | GTGGGCGACGGGCTTATCTG | qPCR |
| IL8-F | TGGCAGTTTTCCTGCTTTCT | qPCR |
| IL8-R | CAGTGGGGTCCACTCTCAAT | qPCR |
| IL1β-F | GGCTCTCCACCTCCTCACAG | qPCR |
| IL1β-R | CTTTGGGTATCTTTTGGGGTCTAT | qPCR |
| IL1α-F | CAAGGACAGTGTGGTGATGG | qPCR |
| IL1α-R | TCATGTTGCTCTGGAAGCTG | qPCR |
| IL6-F | TGGCTACTGCCTTCCCTACC | qPCR |
| IL6-R | CAGAGATTTTGCCGAGGAT | qPCR |
| EPHA4-F | CCCCTGGAGGTCACAACTAA | qPCR |
| EPHA4-R | CGTCTCCTGCTGATGACAAA | qPCR |
| APP-F | GTGAAGATGGATGCGGAGTT | qPCR |
| APP-R | GTGATGACAATCACGGTTGC | qPCR |
| ID2-F | ACTCGCACCCCACTATTGTC | qPCR |
| ID2-R | AATTCAGAAGCCTGCAAGGA | qPCR |
| GAPDH-F | ATCCCGCCAACATCAAAT | qPCR |
| GAPDH-R | TCACGCCCATCACAAACAT | qPCR |
| EGR1-F | AGGTCACCATGGAAGGTCTG | qPCR |
| EGR1-R | TCCAAAATCCATGCAAATCA | qPCR |
| EGR1-sgRNA-1-F | GCTGTTACCGCCGCTGCCCT | Knockout |
| EGR1-sgRNA-1-R | AGGGCAGCGGCGGTAACAGC | Knockout |
| EGR1-sgRNA-2-F | GCCACAGCTTACCTGCGGTC | Knockout |
| EGR1-sgRNA-2-R | GACCGCAGGTAAGCTGTGGC | Knockout |
| EGR1-ID-F | GGCCCACCCGCCCCAACAC | KO PCR |
| EGR1-ID-R | GCACCGCTAAGGACGCCGAGGAT | KO PCR |
